# Radiomics approach to the condylar head for legal age classification using cone-beam computed tomography: A pilot study

**DOI:** 10.1371/journal.pone.0280523

**Published:** 2023-01-19

**Authors:** Kug Jin Jeon, Young Hyun Kim, Hanseung Choi, Eun-Gyu Ha, Hui Jeong, Sang-Sun Han

**Affiliations:** Department of Oral and Maxillofacial Radiology, Yonsei University College of Dentistry, Seoul, Republic of Korea; University of the Pacific Arthur A Dugoni School of Dentistry, UNITED STATES

## Abstract

Legal age estimation of living individuals is a critically important issue, and radiomics is an emerging research field that extracts quantitative data from medical images. However, no reports have proposed age-related radiomics features of the condylar head or an age classification model using those features. This study aimed to introduce a radiomics approach for various classifications of legal age (18, 19, 20, and 21 years old) based on cone-beam computed tomography (CBCT) images of the mandibular condylar head, and to evaluate the usefulness of the radiomics features selected by machine learning models as imaging biomarkers. CBCT images from 85 subjects were divided into eight age groups for four legal age classifications: ≤17 and ≥18 years old groups (18-year age classification), ≤18 and ≥19 years old groups (19-year age classification), ≤19 and ≥20 years old groups (20-year age classification) and ≤20 and ≥21 years old groups (21-year age classification). The condylar heads were manually segmented by an expert. In total, 127 radiomics features were extracted from the segmented area of each condylar head. The random forest (RF) method was utilized to select features and develop the age classification model for four legal ages. After sorting features in descending order of importance, the top 10 extracted features were used. The 21-year age classification model showed the best performance, with an accuracy of 91.18%, sensitivity of 80%, and specificity of 95.83%. Radiomics features of the condylar head using CBCT showed the possibility of age estimation, and the selected features were useful as imaging biomarkers.

## Introduction

Age estimation of living individuals, particularly the estimation of legal age, is a critically important issue that needs to be addressed on a global scale [1]. Some challenges, such as the increasing number of international refugees as a result of conflict and legal disputes regarding criminal culpability, exacerbate the necessity for age assessment [2]. Physical examinations and facial photographs are often utilized as easily accessible information to estimate the age of a living individual [3–5]. Anatomical structures such as the skull, mandible, and teeth, as well as hands, are also considered to be powerful tools for age estimation [1, 6–8]. Since no age estimation methods are guaranteed to be 100% accurate, novel approaches for age estimation need to be developed.

The shape of the mandible is closely related to age, enabling it to be used as a potential indicator for age estimation [9]. Bayrak et al. [10] reported a correlation between chronologic age and mandibular condyle cortication through a visual assessment of cone-beam computed tomography (CBCT) scans. The conversion of bone marrow in the mandible according to age was observed on magnetic resonance imaging (MRI), and the presence of recognizable red marrow in the condylar head decreases around the ages of 25 to 30 [11]. Previous studies have suggested the potential of bone marrow in the condylar head as an imaging biomarker for age estimation, but no studies have specifically been conducted on age classification using this parameter.

Radiomics is an emerging research field that extracts quantitative data from medical images using data-characterization algorithms [12]. This approach allows experts to obtain more diagnostic information from medical images, which are considered an important decision-making tool for personalized care [13, 14]. Some studies have used CBCT images to implement a radiomics approach with the mandibular condylar head [15–17]. These efforts have demonstrated the feasibility of radiomics features as biomarkers for diagnosing bony changes of the mandibular condyle. However, no reports have proposed age-related radiomics features of the condylar head or an age classification model using those features.

Legal ages vary from 14 to 21 years depending on the country or law, but most are in the range of 18 to 21 years. Therefore, the purpose of this pilot study was to introduce a radiomics approach for legal age classification (18, 19, 20, and 21 years old) based on CBCT images of the mandibular condylar head, and to assess the usefulness of the selected features as an imaging biomarker using machine learning models.

## Materials and methods

### Subjects

A total of 170 condylar heads from 85 subjects (age range from 10 to 25 years, 43 males and 42 females) who underwent CBCT at Yonsei University Dental Hospital between September 2017 and November 2020 were retrospectively selected. The inclusion criteria were as follows: subjects who had CBCT for reasons other than temporomandibular disorder, no pathologic bony changes on the mandibular condylar head, and no significant image artifacts affecting radiomic parameters.

All CBCT images were exported after de-identifying subject information in the picture archiving and communication system. Alphard 3030 devices (Asahi Roentgen Ind., Co., Ltd, Tokyo, Japan) were used to obtain CBCT data with settings of 80 kVp, 8 mA, 154 × 154 mm^2^ field of view, 0.3 mm^3^ voxel size, and an exposure time of 17 s. The mandibular condyles of the subjects were treated individually and separated into eight groups based on age for four legal age classifications: ≤17 and ≥18 years old groups (18-year age classification), ≤18 and ≥19 years old groups (19-year age classification), ≤19 and ≥20 years old groups (20-year age classification) and ≤20 and ≥21 years old groups (21-year age classification). For example, if a subject was 18 years old, he or she would be included in the ≥18 years old group, ≤19 years old group, ≤20 years old group and ≤21 years old group, respectively.

### Image segmentation and radiomics feature extraction

**Fig 1:**
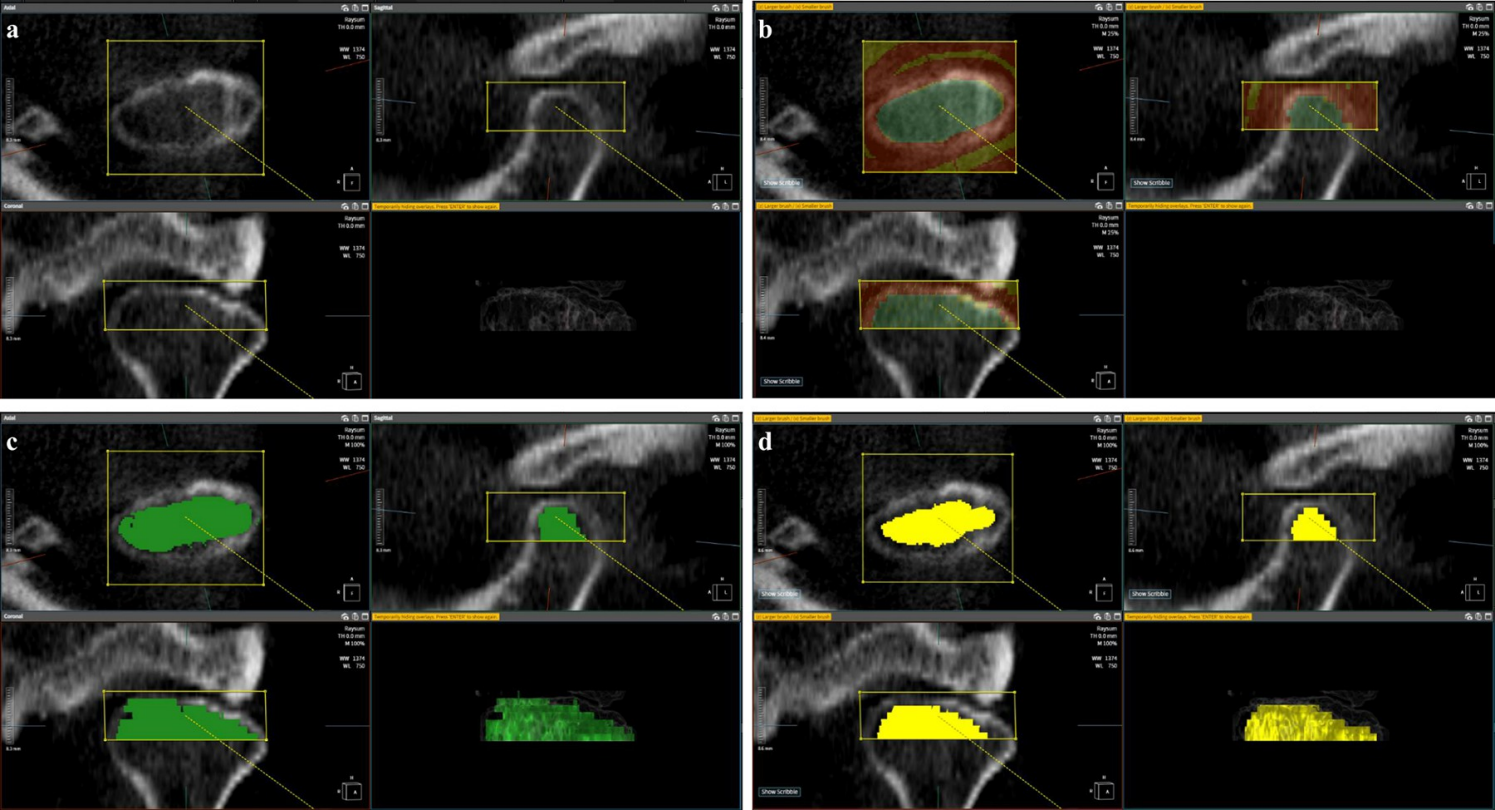
Three-dimensional (3D) volume of interest (VOI) on mandibular condylar head. (a) Drawing of a yellow box to fit the condylar head on axial, sagittal, and coronal images; (b) drawing of the VOI area as green and non-VOI area as red on 2–3 sections; (c) gross 3D segmentation results after the magic cut function of AVIEW research software version 34.35 (Coreline Soft Inc., Seoul, Korea) (green), (d) final 3D VOI after radiologist correction (yellow).

CBCT data were imported into the AVIEW research software version 34.35 (Coreline Soft Inc., Seoul, Korea). Reconstructed axial, sagittal, and coronal views along the long axis of the condylar head were used. The three-dimensional volume of interest (VOI) of the condylar head was established along the inner surface of the cortical layer above the longest axis of the condylar head on the coronal image for radiomics feature extraction. An oral and maxillofacial radiologist with 20 years of experience performed manual segmentation using the magic cut function of AVIEW on axial, sagittal, and coronal images. The radiologist set up a yellow square box including the condylar head and drew the VOI area as green and non-VOI area as red on 2–3 images, and the magic cut function then automatically segmented the condyle on all images. The automatic segmentation of the condyle was reviewed and corrected by the radiologist to obtain the final 3D VOI (Fig 1).

In the AVIEW software, 127 radiomics features were calculated for the segmented area of the condylar head. The radiomics features consisted of 1 fractal feature, 23 shape and size features, and 103 texture features. Texture features provide various kinds of statistical information about gray-value distribution, and there are 10 categories: first order features, histogram features, percentiles, gradient features, gray-level co-occurrence matrix (GLCM) features, gray-level run-length matrix (GLRLM) features, gray-level size zone matrix (GLSZM) features, neighboring gray-tone difference matrix (NGTDM) features, gray-level dependence matrix (GLDM) features, and moment features. All 127 feature definitions and categories are provided in the AVIEW reference manual (S1 File).

To assess the reliability of the segmentation process, the radiologist performed 3D VOI segmentation process twice on 32 randomly selected condylar heads and extracted the values of radiomics features. The intraclass correlation coefficient (ICC) was calculated between pairs of extracted values and showed excellent agreement (ICC = 0.968).

### Important feature selection and age classification model development

Random forest (RF) was used for radiomics feature selection and age classification model. RF is one of the machine learning methods for discrete variables and is an ensemble classifier that produces multiple decision trees using a randomly selected subset of training samples and variables [18]. It calculates the importance of input variables based on Gini impurity for feature selection [19]. We conducted radiomics feature selection with the top 10 features for 18, 19, 20 and 21-year age classifications, respectively. The top 10 features were 10 features extracted when sorting in descending order of feature importance in the RF models. The process for feature selection and age classification model development was implemented using Python (version 3.7.13) and the Scikit-learn package (version 1.0.2) [20]. The selected top 10 features for each age classification are listed in Table 1.

**Table 1:** Selected top 10 features in the random forest model for 18, 19, 20 and 21-year age classifications.

	18-year age classification	19-year age classification	20-year age classification	21-year age classification
**Texture features**	Grad_Std	Grad_Std	Grad_Std	Grad_Std
	GLCM_Contrast	GLCM_DiffVariance	GLCM_Homogeneity	Grad_Mean
	GLCM_DiffAverage	GLCM_ASM	FirstOrder_Min	GLCM_DiffVariance
	GLCM_ASM	GLCM_IDMN	FirstOrder_Max	GLCM_Homogeneity
	GLCM_IDM	GLRLM_RP	FirstOrder_RMS	GLCM_IDMN
	GLCM_Entropy	GLRLM_LRLGE	FirstOrder_TotalEnergy	GLCM_Contrast
	GLCM_Homogeneity	FirstOrder_Max	Percentile_25	GLDM_SDHGLE
	GLRLM_RE	NGTDM_Strength	Percentile_50	GLSZM_LAHGLE
	GLRLM_RV		Percentile_75	FirstOrder_RMS
	GLRLM_SRHGE			
**Shape and size features**		SphericalDisproportion	Compactness2	Longest2ndAxisOnCoronal(mm)
		Flatness		
**Fractal feature**				

Top 10 features extracted when sorting in descending order of feature importance in the random forest. All feature definitions are provided in the AVIEW reference manual (S1 File).

Abbreviations: GLCM, gray level cooccurrence matrix; ASM, angular second momentum; IDMN, inverse difference moment normalized; IDM, inverse difference moment; GLRLM, gray level run length matrix; RP, run percentage; RMS, root mean squared; LRLGE, long run low gray level emphasis; RE, run entropy; RV, run variance; SRHGE, short run high gray level emphasis; GLDM, gray level dependence matrix; SDHGLE, small dependence high gray level emphasis; NGTDM, neighbouring gray tone difference matrix; GLSZM, gray level size zone matrix; LAHGLE, large area high gray level emphasis.

For the development of the age classification model, we entered selected features in the RF models. The number of decision trees (n_estimator) and the maximum depth of decision trees (max_depth), which are parameters of the tree-based models, were set to the values with the highest prediction accuracy by sequentially assigning them from 1 to 500 and from 1 to 10, respectively. The training and test datasets were randomly assigned at a ratio of 8:2 (Table 2). 78 subjects were used in the training datasets and 17 subjects were used in the test datasets. Four age classification models were built with the training dataset, and the test dataset was applied to verify the model performance.

**Table 2:** Number of database distributions according to the age classification model.

Groups	18-year age classification		19-year age classification		20-year age classification		21-year age classification	
	Training	Test	Training	Test	Training	Test	Training	Test
**Male**	68	18	68	18	68	18	68	18
**Female**	68	16	68	16	68	16	68	16
**Total**	170		170		170		170	

Prediction model performance was evaluated in terms of accuracy, sensitivity, and specificity for the four age classification models, which are defined as:

$$Accuracy=\frac{TP+TN}{TP+TN+FP+FN}$$ (1)

$$Sensitivity=\frac{TP}{TP+FN}$$ (2)

$$Specificity=\frac{TN}{TN+FP}$$ (3)

where TP, TN, FP, and FN are true positive, true negative, false positive, and false negative, respectively.

### Ethics approval

Institutional Review Board (IRB) approval was obtained from Yonsei University Dental Hospital (IRB No. 2-2021-0086) to conduct this study. The requirement for patients’ informed consent was waived by this ethics committee because of the retrospective nature of the study. All CBCT images were anonymized and used in accordance with the relevant guidelines and ethical regulations.

## Results

Table 3 shows the performance of the RF models for four age classifications. The 21-year age classification model showed an accuracy of 91.18%, sensitivity of 80%, and specificity of 95.83%.

**Table 3:** Accuracy, sensitivity, and specificity of the four age classification models.

	Groups	18-year age classification	19-year age classification	20-year age classification	21-year age classification
**Accuracy (%)**	**Male**	72.22	72.22	88.89	88.89
	**Female**	100	93.75	75	93.75
	**Total**	85.29	82.35	82.35	91.18
**Sensitivity (%)**	**Male**	75	62.5	83.33	66.67
	**Female**	100	83.33	83.33	100
	**Total**	87.50	71.43	83.33	80.00
**Specificity (%)**	**Male**	70	80	91.67	100
	**Female**	100	100	70	91.67
	**Total**	83.33	90.00	81.82	95.83

**Fig 2:**
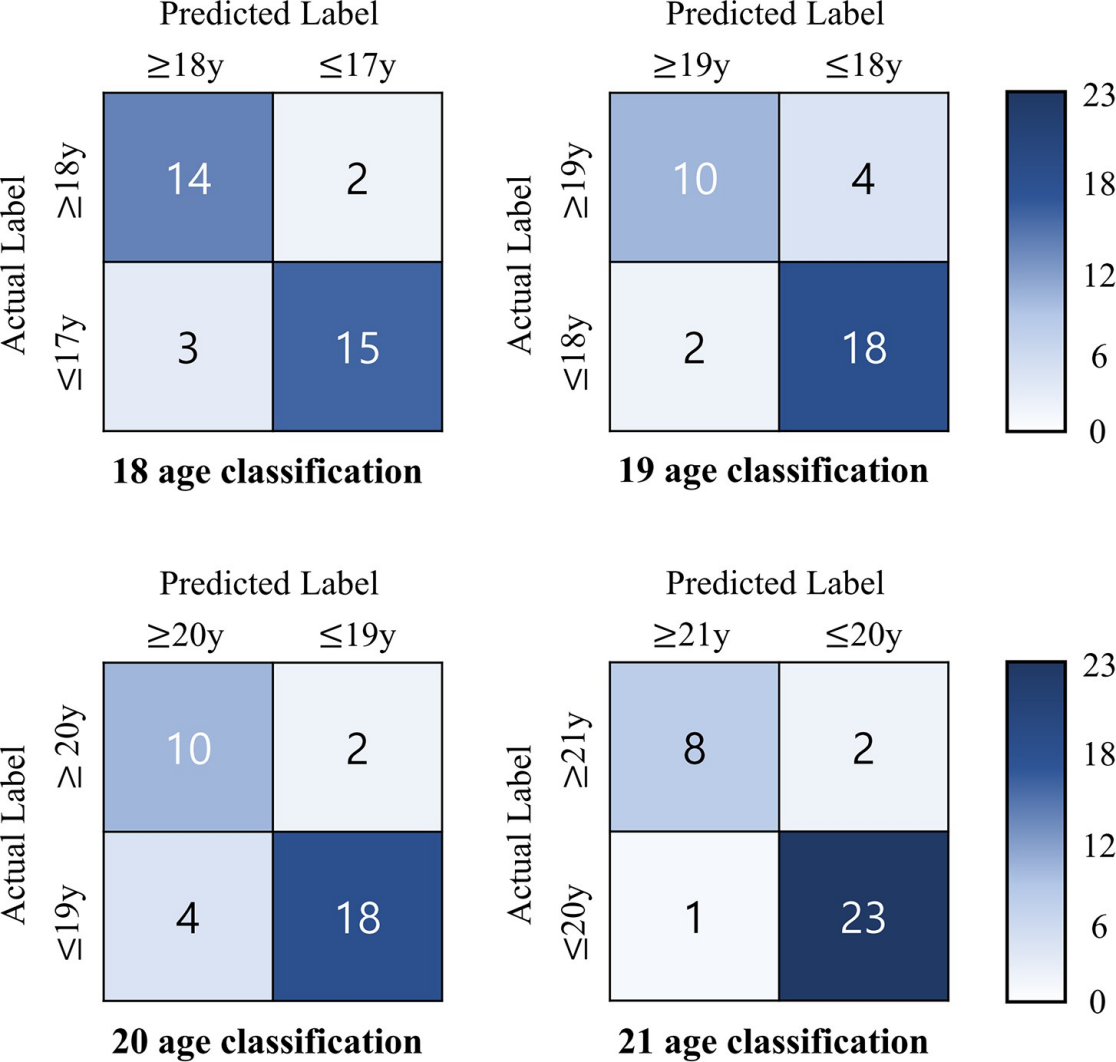
Confusion matrices of the four age classification models.

Fig 2 indicates the 2×2 confusion matrices of the models. Of the 34 scans used for testing, the 21-year age classifier model correctly predicted 31 condylar heads.

## Discussion

In the present study, a machine learning model was proposed to classify various legal ages using radiomics features of the mandibular condylar head based on CBCT. Various methods are used for age estimation, and studies using radiography as a less-invasive modality in living people are being conducted. Most studies using radiography have utilized panoramic radiography, although the number of studies using CBCT, which can be used to perform 3D analyses and has a relatively low radiation dose compared to computed tomography (CT), is increasing. However, most studies using CBCT analyzed tooth development or the pulp/tooth ratio [21–23]. Many researchers have estimated age using body parts such as the skull, teeth, hand wrist, and cervical spine, whereas studies using the mandibular condyle are rare. Bayrak et al. [10] studied the relationship between condylar cortication and chronologic age in CBCT scans of 433 patients aged 8 to 31 years, and reported that cortication increased with age. Kaneda et al. [11] reported that bone marrow conversion in the mandible from red marrow to yellow marrow was observed first in the premolar/molar region, followed by the angle, ramus, and condyle region with increasing age, using MRI. They suggested that age-related bone marrow conversion could be useful for differentiating abnormal bone marrow in conditions such as anemia, inflammatory disease, invasive tumor disease, and metastatic disease.

Radiomics provides an objective and quantitative method to assess disease, and has shown promise for differentiating benign and malignant tumors and predicting the therapeutic response of malignant tumors [24]. Rastegar et al. [25] reported a high area under the curve (AUC) of 0.78 in a machine learning model for distinguishing between the normal state and osteoporosis in the lumbar and femoral regions. The usability of a machine learning model for the early diagnosis of osteoarthritis in the condylar head was reported; when applied to 92 CBCT images, the model showed an accuracy of 0.823, an AUC of 0.870, and an F1-score of 0.823 [26]. However, no study has yet used radiomics for age estimation. We used the RF method for training and testing the radiomics model. Depending on whether the outcome of the clinical question is a continuous variable or a discrete variable, Papanikolaou et al. [27] reported that regression methods such as linear regression, Cox (proportional hazards) models, and regression trees can be used for continuous variables. For discrete variables, classification methods such as RF, K-nearest neighbors, naive Bayes, support vector machines, and decision trees can be used. Overfitting and the excessive use of computer resources have occurred when multiple features are employed to build predictive models using radiomics; therefore, various techniques for selecting critical features have been suggested [28]. In our study, only 10 major features were finally selected from the initial 127 radiomics features. By using the top 10 radiomics features selected from the RF model, we reduced overfitting and developed a more generalized and simpler predictive model.

The radiomics features extracted by manually segmenting the condyle twice by one radiologist were reliable, with ICC of 0.968. The accuracy of the 21-year age classification model was the highest at 91.18%, and the accuracy and specificity exceeded 82% and 81% for all legal age classification models. Cameriere et al. [29] evaluated the legal adult age of 18 years by measuring open apices of the third molar on panoramic radiography. The ICC was 0.998 and the accuracy was 87.5%. Compared with legal age classification using the commonly used third molar development, our 21-year age classification showed higher accuracy. For model performance according to sex, accuracy and sensitivity in females were higher than in males, except for 20-year age classification. The models demonstrated the possibility for age estimation, but the sensitivity showed a lower value. In males, the sensitivity of the 19- and 21-year age classifications was less than 70%. Therefore, additional research is needed to improve the sensitivity of the model and to develop a model according to sex in the future.

This study has several limitations. First, the number of samples was small and external validation was not performed. Second, it would be difficult to apply this model to subjects with bony changes on the condylar head due to disease. In future research, it is expected that more data will be used to improve the accuracy, especially sensitivity, of the prediction model, and various age estimation and classification tasks based on a radiomics approach will be attempted.

## Conclusions

We conducted a pilot study to determine individuals’ age classification through a radiomics approach using the condylar head in CBCT images. The accuracy of the developed classification model demonstrated satisfactory performance and the selected features as an imaging biomarker using machine learning models were useful. The radiomics of the condyles using CBCT showed potential for use in age classification.

## Supporting information

S1 File Radiomics features in AVIEW research. Republished from PLOS Journal under a CC BY license, with permission from Coreline Soft, original copyright [2021].
(PDF)

S1 Data (PDF)
