# Supplementary material for: Radiomics approach to the condylar head for legal age classification using cone-beam computed tomography: A pilot study
Source: PLoS One. 2023 Jan 19;18(1):e0280523. doi: 10.1371/journal.pone.0280523 (PMC9851527; doi:10.1371/journal.pone.0280523)

Radiomics Features in AVIEW Research

Rev. 1.0

코어라인소프트
CORELINE SOFT

Copyright Notification

© 2012-2019 Coreline Soft. All rights are reserved. This publication and its contents are proprietary to Coreline Soft. No part of this publication may be reproduced in any form or by any means without the written permission of Coreline Soft.

Revision History

Revision	Date	Author	Changes
0.1	2018.04.29	Jaeyoun Yi	Initially wrote. (Based on 1.0.22.6)
0.2	2018.05.02	Hyungi Seo	Corrected 'Shape_Sphericity' formula.
0.3	2018.06.27	Jaeyoun Yi	Added 2D shape features. Changed 'Compactness'
0.4	2019.01.30	Donghoon Yu	Added multiple number of features.
0.5	2019.02.15	Seongeun Ahn	Added more description on GLCM and GLCM_IMC1. Fixed typos in the description.
0.6	2019.02.20	Donghoon Yu	Changed formula (Changed 1-base index to 0-base index)
0.7	2019.03.25	Jaeyoun Yi	Translated to English
0.8	2019.04.08	Donghoon Yu	Added normalization method.
0.9	2019.04.28	Donghoon Yu	Fixed the normalization equation.
0.91	2019.06.27	Jaeyoun Yi	Revised the document.
1.0	2021.03.17	Donghoon Yu	Added an example at GLRLM

Contents

REVISION HISTORY 1

1 INTRODUCTION 3

1.1 LIST OF FEATURES 3

2 TEXTURE FEATURES 9

2.1 CAUTION ABOUT PARAMETERS 9

2.2 NORMALIZATION 11

2.3 FIRST ORDER FEATURES 11

2.4 HISTOGRAM FEATURES 17

2.5 PERCENTILE VALUES 19

2.6 GRADIENT FEATURES 21

2.7 GLCM FEATURES (HARALICK FEATURES) 22

2.8 GLRLM FEATURES 29

2.9 GLSZM FEATURES 34

2.10 NGTDM FEATURES 40

2.11 GLDM FEATURES 43

2.12 MOMENT FEATURES 48

3 SHAPE FEATURES 49

3.1 CAUTIONS 49

3.2 SHAPE FEATURES IN 3D-VIEW 49

3.3 SHAPE FEATURES IN 2D-VIEW 54

4 FRACTAL FEATURES 57

4.1 CAUTIONS 57

4.2 FRACTAL FEATURES 57

1 Introduction

This document is based on AVIEW version 1.0.25.9.

1.1 List of features

For a single lesion VOI, following features are calculated.

First Order Features (14 features)

- Texture_FirstOrder_Energy
- Texture_FirstOrder_TotalEnergy
- Texture_FirstOrder_Min
- Texture_FirstOrder_Max
- Texture_FirstOrder_Range
- Texture_FirstOrder_Mean
- Texture_FirstOrder_MAD
- Texture_FirstOrder_rMAD
- Texture_FirstOrder_RMS
- Texture_FirstOrder_Std
- Texture_FirstOrder_Skewness
- Texture_FirstOrder_ExcessKurtosis
- Texture_FirstOrder_Variance
- Texture_FirstOrder_InterquartileRange

Histogram Features (9 features)

- Texture_Histo_Mean
- Texture_Histo_Std
- Texture_Histo_Skewness
- Texture_Histo_ExcessKurtosis
- Texture_Histo_Energy
- Texture_Histo_Entropy
- Texture_Histo_Min
- Texture_Histo_Max
- Texture_Histo_VoxelCount

Percentiles (6 percentiles)

- Texture_Percentile_10
- Texture_Percentile_25
- Texture_Percentile_50
- Texture_Percentile_75
- Texture_Percentile_90
- Texture_Percentile_95

Gradient Features (2 features)

- Texture_Grad_Mean
- Texture_Grad_Std

GLCM Features (22 features)

- Texture_GLCM_ASM
- Texture_GLCM_IDM
- Texture_GLCM_IDMN
- Texture_GLCM_Homogeneity
- Texture_GLCM_HomogeneityNormalized
- Texture_GLCM_InverseVariance
- Texture_GLCM_Contrast
- Texture_GLCM_Correlation
- Texture_GLCM_Autocor
- Texture_GLCM_Entropy
- Texture_GLCM_CP
- Texture_GLCM_CS
- Texture_GLCM_CT
- Texture_GLCM_SumEntropy
- Texture_GLCM_DiffAverage,
- Texture_GLCM_DiffEntropy
- Texture_GLCM_DiffVariance
- Texture_GLCM_IMC1
- Texture_GLCM_IMC2
- Texture_GLCM_MCC

- Texture_GLCM_MaxProb
- Texture_GLCM_SumAverage

GLRLM Features (14 features)

- Texture_GLRLM_SRE
- Texture_GLRLM_LRE
- Texture_GLRLM_LGRE
- Texture_GLRLM_HGRE
- Texture_GLRLM_SRLGE
- Texture_GLRLM_SRHGE
- Texture_GLRLM_LRLGE
- Texture_GLRLM_LRHGE
- Texture_GLRLM_GNUN
- Texture_GLRLM_RLNUN
- Texture_GLRLM_RP
- Texture_GLRLM_RV
- Texture_GLRLM_RE
- Texture_GLRLM_GLV

GLSZM Features (16 features)

- Texture_GLSZM_SAE
- Texture_GLSZM_LAE
- Texture_GLSZM_GLN
- Texture_GLSZM_GLNN
- Texture_GLSZM_SZN
- Texture_GLSZM_SZNN
- Texture_GLSZM_ZP
- Texture_GLSZM_GLV
- Texture_GLSZM_ZV
- Texture_GLSZM_ZE
- Texture_GLSZM_LGLZE
- Texture_GLSZM_HGLZE
- Texture_GLSZM_SALGLE
- Texture_GLSZM_SAHGLE

- Texture_GLSZM_LALGLE
- Texture_GLSZM_LAHGLE

NGTDM Features (5 features)

- Texture_NGTDM_Coarseness
- Texture_NGTDM_Contrast
- Texture_NGTDM_Busyness
- Texture_NGTDM_Complexity
- Texture_NGTDM_Strength

GLDM Features (14 features)

- Texture_GLDM_SDE
- Texture_GLDM_LDE
- Texture_GLDM_GLN
- Texture_GLDM_DN
- Texture_GLDM_DNN
- Texture_GLDM_GLV
- Texture_GLDM_DV
- Texture_GLDM_DE
- Texture_GLDM_LGLE
- Texture_GLDM_HGLE
- Texture_GLDM_SDLGLE
- Texture_GLDM_SDHGLE
- Texture_GLDM_LDLGLE
- Texture_GLDM_LDHGLE

Moment Features (3-features)

- Texture_Moment_J1
- Texture_Moment_J2
- Texture_Moment_J3

Shape Features (23-features) in 3D-view

- Shape3D_Volume(mm3)
- Shape3D_SurfaceArea(mm2)

- Shape3D_SurfaceAreaToVolumeRatio
- Shape3D_Sphericity
- Shape3D_Compactness
- Shape3D_Compactness2
- Shape3D_Compactness3
- Shape3D_Roundness
- Shape3D_Circularity
- Shape3D_SphericalDisproportion
- Shape3D_Longest1stAxis(mm)
- Shape3D_Longest2ndAxis(mm)
- Shape3D_Longest1stAxisOnAxial(mm)
- Shape3D_Longest2ndAxisOnAxial(mm)
- Shape3D_Longest1stAxisOnSagittal(mm)
- Shape3D_Longest2ndAxisOnSagittal(mm)
- Shape3D_Longest1stAxisOnCoronal(mm)
- Shape3D_Longest2ndAxisOnCoronal(mm)
- Shape3D_PCA1stMajorStd(mm)
- Shape3D_PCA2ndMajorStd(mm)
- Shape3D_PCA3rdMajorStd(mm)
- Shape3D_Elongation
- Shape3D_Flatness

Shape Features (11-features) in 2D-view

- Shape2D_Area(mm2)
- Shape2D_Perimeter(mm)
- Shape2D_PerimeterToAreaRatio
- Shape2D_Circularity
- Shape2D_Compactness
- Shape2D_Roundness
- Shape2D_LongestAxis(mm)
- Shape2D_OrthogonalAxis(mm)
- Shape2D_PCAMajorStd(mm)
- Shape2D_PCAMinorStd(mm)
- Shape2D_Flatness

Fractal Features (1 feature)

- FractalDimension

Refer to the following chapters for the detailed explanation.

2 Texture Features

Texture features comprise the various kinds of statistical information about the gray-value distribution inside the VOI box. If the lesion VOI has a segmentation mask, all the texture features are calculated inside the mask only.

2.1 Caution about parameters

Some parameters should be pre-defined to calculate texture features. Different parameters could produce different values of texture features. Therefore, you need to decide the parameters before you begin your research carefully, and keep the values be fixed throughout your whole research period. If you change any of the following parameters during your research, you must re-calculate all the features again.

MaxPixelValue, MinPixelValue

These define the minimum and the maximum values of gray-values. The gray values outside of these values shall be truncated to the range. These 2 values are applied to histogram features, GLCM, GLSZM, GLRLM, NGTDM, and GLDM. It is recommended to set these values to include the valid gray-value range of organs of your interest. One of the useful recommendations is to refer the WWL (Window Width & Level) values in PACS viewers.
These values are not relevant to 'First order features,' 'shape features,' and 'fractal features.'

Default: -1000 (MinPixelValue), 1000 (MaxPixelValue)

GLCMBins

GLCM is a short term for 'Gray-Level Co-occurrence Matrix,' which is a well-known method to analyze the repeating pattern of gray-levels. To determine if two voxels have the same gray-value, the gray values are quantized into 'GLCMBins' bins between MinPixelValue and MaxPixelValue.
If you set this value too small, the algorithm can't differentiate the gray values. If you set this value too high, the algorithm may be sensitive to noise. Also, it should be noted that higher value increases the computational complexity so that the calculation time takes longer.

Default: 64

GLRLMBins

GLRLM is a short term for 'Gray-Level Run-Length Matrix'. It quantifies gray-level runs, which are defined as the length of consecutive voxels that have the same gray level value. To determine if two voxels have the same gray-value, the gray values are quantized into 'GLRLMBins' bins between MinPixelValue and MaxPixelValue.
If you set this value too small, the algorithm can't differentiate the gray values. If you set this value too high, the algorithm may be sensitive to noise. Also, it should be noted that higher value increases the computational complexity so that the calculation time takes longer.

Default: 64

GLSZMBins

GLSZM is a short term of 'Gray-Level Size Zone Matrix'. It quantifies gray level zones in an image. A gray level zone is defined as the number of connected voxels that share the same gray level intensity. A voxel is considered connected if the distance is 1, according to the infinity norm (26 connectivity). To determine if two voxels have a same gray-value, the gray values are quantized into 'GLRLMBins' bins between MinPixelValue and MaxPixelValue.
If you set this value too small, the algorithm can't differentiate the gray values. If you set this value too high, the algorithm may be sensitive to noise. Also, it should be noted that higher value increases the computational complexity so that the calculation time takes longer.

Default: 64

NGTDMBins

NGTDM is a short term for 'Neighbouring Gray-Tone Difference Matrix.' It quantifies the difference between a gray value and the average gray value of its neighbors within a predefined distance (We use 1 for the distance value). The sum of absolute differences for the gray level is stored in the matrix. To determine if two voxels have the same gray-value, the gray values are quantized into 'GLRLMBins' bins between MinPixelValue and MaxPixelValue.
If you set this value too small, the algorithm can't differentiate the gray values. If you set this value too high, the algorithm may be sensitive to noise. Also, it should be noted that higher value increases the computational complexity so that the calculation time takes longer.

Default: 64

GLDMBins

GLDM is a short term for 'Gray-Level Dependence Matrix.' It quantifies gray level dependencies in an image. A gray level dependency is defined as the number of connected voxels within a predefined distance (We use 1 for the distance value.) To determine if two voxels have the same gray-value, the gray values are quantized into 'GLRLMBins' bins between MinPixelValue and MaxPixelValue.

If you set this value too small, the algorithm can't differentiate the gray values. If you set this value too high, the algorithm may be sensitive to noise. Also, it should be noted that higher value increases the computational complexity so that the calculation time takes longer.

Default: 64

2.2 Normalization

The intensity values could be normalized by setting `NormalizeIntensity` parameter as `Yes'. Normalization is based on gray values in the whole image (or volume), not inside the segmented image (or volume) only. This setting affects the intensity based features only.

$$f(x) = s \cdot \frac{x - \mu_x}{\sigma_x},$$

Where, x and $f(x)$ are the original and normalized intensity, respectively. μ_x and σ_x is the mean and the standard deviation of the image intensity values, and s is a scaling defined by `NormalizedIntensityScale` parameter.

Outliers are removed, in which case values for which $\mathrm{x} > \mu_x + n\sigma_x$ or $\mathrm{x} < \mu_x - n\sigma_x$ are set to $\mu_x + n\sigma_x$ and $\mu_x - n\sigma_x$, respectively. μ_x is the mean of the intensity values, and n is defined by `NormalizeIntensityOutlier` parameter.

Default:

- NormalizeIntensity: No
- NormalizeIntensityScale: 5000
- NormalizeIntensityOutlier: 6.0

2.3 First Order Features

These features describe the distribution of voxel intensities inside a VOI. If the VOI has a mask, the 1st order statistics values are calculated inside the mask only. It is noted that these values are not dependent on MinPixelValue and

MaxPixelValue parameters.

Texture_FirstOrder_Energy

Energy

It is a measure of the magnitude of voxel values. A larger value implies a greater sum of the squares of the gray values.

$$\text{Energy} = \sum_{i=0}^{N-1} (x_i + c)^2$$

, where x_i is the voxel value at each location, N is the number of voxels inside the VOI, and c shifts the intensity to prevent negative values. The value of c is set to 1024 for CT modality, and 0 for other modalities.

Texture_FirstOrder_TotalEnergy

Total energy

It is the value of Energy feature scaled by the volume of the voxels in mm^3.

$$\text{Total energy} = V_{voxel} \sum_{i=0}^{N-1} (x_i + c)^2$$

, where V_{voxel} is the volume of a voxel (mm^3)

Texture_FirstOrder_Min

Minimum value

The minimum voxel gray value inside the VOI.

$$\text{Minimum} = \min(\text{X})$$

Texture_FirstOrder_Max

Maximum value

The maximum voxel gray value inside the VOI.

$$\text{Maximum} = \max(X)$$

Texture_FirstOrder_Range

Range

The range of gray values in the VOI.

$$\text{Range} = \max(X) - \min(X)$$

Texture_FirstOrder_Mean

Mean

The average of gray level intensities within the VOI.

$$\mu = \mathrm{E}[X] = \frac{\sum x_i}{N}$$

, where x_i is the voxel value at each location and N is the number of voxels inside the VOI.

Texture_FirstOrder_MAD

Mean absolute deviation

It is the mean distance of all intensity values from the 'Mean'.

$$MAD = \frac{1}{N}\sum_{i=0}^{N-1}|x_i - \mu|$$

, where x_i is the voxel value at each location, N is the number of voxels inside the VOI and μ is the mean of the VOI.

Texture_FirstOrder_rMAD

Robust mean absolute deviation

It is similar to 'Mean absolute deviation', but it excludes odd values outside 10th and 90th percentiles.

$$rMAD = \frac{1}{N_{10-90}} \sum_{i=0}^{N_{10-90}-1} |x_i - \mu_{10-90}|$$

, where $N_{10\text{-}90}$ is the number of voxels in between or equal to the 10th and 90th percentile and μ is the mean of them.

Texture_FirstOrder_RMS

Root mean squared

It is another measure of the magnitude of the image values.

$$RMS = \sqrt{\frac{1}{N}\sum_{i=0}^{N-1}(x_i + c)^2}$$

, where x_i is the voxel value at each location, N is the number of voxels inside the VOI, and c shifts the intensity to prevent negative values. The value of c is set to 1024 for CT modality, and 0 for other modalities.

Texture_FirstOrder_Std

Standard deviation

Amount of variation or dispersion from the 'Mean' value.

$$\sigma = \sqrt{\mathrm{E}[(X-\mu)^2]} = \sqrt{\frac{1}{N}\sum_{i=0}^{N-1}(x_i - \mu)^2}$$

, where x_i is the voxel value at each location, N is the number of voxels inside the VOI and μ is the mean of the VOI.

Texture_FirstOrder_Skewness

Skewness

It measures the asymmetry of the distribution of gray values about the 'Mean' value.

$$\gamma_1 = \mathrm{E}\left[\left(\frac{X-\mu}{\sigma}\right)^3\right] = \frac{\mathrm{E}[X^3] - 3\mu\,\mathrm{E}[X^2] + 2\mu^3}{\sigma^3} = \frac{\mathrm{E}[X^3] - 3\mu\sigma^2 - \mu^3}{\sigma^3}$$

, where μ and σ are the mean and standard-deviation of the VOI, respectively.

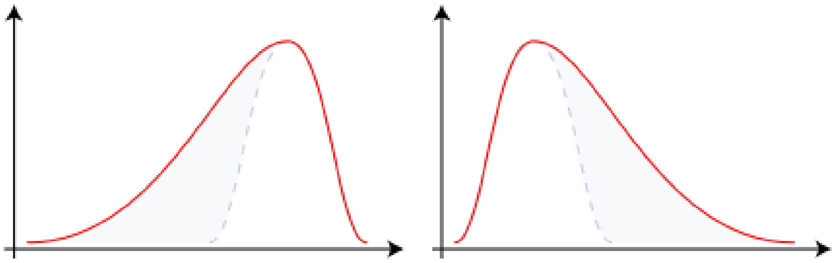

Texture_FirstOrder_ExcessKurtosis

Excess Kurtosis

Kurtosis is a measure of the 'peakedness' of the distribution of gray values. Excess Kurtosis is simply defined as 'Kurtosis – 3'. If the value is close to zero, the distribution looks similar to normal distribution. When the value is greater than 0, the distribution is steeper than normal distribution. Negative value implies the reverse.

$$\mathrm{ExcessKurt}[X] = \mathrm{E}\left[\left(\frac{X-\mu}{\sigma}\right)^4\right] - 3$$

, where μ and σ are the mean and standard-deviation of the VOI, respectively.

Texture_FirstOrder_Variance

Variance

Variance is the mean of the squared distances of each intensity value. By defitnition, $variance = \sigma^2$

$$\mathrm{Variance} = \mathrm{E}[(X-\mu)^2] = \frac{1}{N}\sum_{i=0}^{N-1}(x_i - \mu)^2$$

, where x_i is the voxel value at each location, N is the number of voxels inside the VOI and μ is the mean of the VOI.

Texture_FirstOrder_InterquartileRange

Interquartile range

$$\text{Interquartile range} = P_{75} - P_{25}$$

, where P_{25} and P_{75} are the 25th and 75th percentile. Refer to 2.5 for more details.

2.4 Histogram Features

Most of the features are very similar to 'First Order Statistics' features. The only difference is that histogram features truncate the gray-values outside the range from 'MinPixelValue' to 'MaxPixelValue.'

Texture_Histo_Mean

Mean

$$\mu = \mathrm{E}[X] = \sum_{b=0}^{B-1} x_b p(b)$$

, where b is the histogram bin index, x_b is the intensity value of index b, and $p(b)$ is the normalized histogram of index b.

Texture_Histo_Std

Standard deviation

$$\sigma = \sqrt{\mathrm{E}[(X-\mu)^2]} = \sqrt{\sum_{b=0}^{B-1} (x_b - \mu)^2 p(b)}$$

, where b is the histogram bin index, x_b is the intensity value of index b, and $p(b)$ is the normalized histogram of index b.

Texture_Histo_Skewness

Skewness

It measures the asymmetry of the distribution of gray values about the 'Mean' value.

$$\mathrm{Skewness} = \mathrm{E}\left[\left(\frac{X-\mu}{\sigma}\right)^3\right] = \sum_{b=0}^{B-1} \left(\frac{x_b-\mu}{\sigma}\right)^3 p(b)$$

, where μ and σ are the mean and standard-deviation of the VOI, respectively.

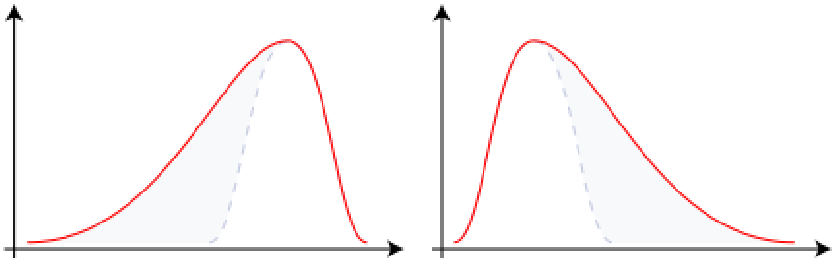

Texture_Histo_ExcessKurtosis

Excess Kurtosis

Kurtosis is a measure of the 'peakedness' of the distribution of gray values. Excess Kurtosis is simply defined as 'Kurtosis – 3'. If the value is close to zero, the distribution looks similar to normal distribution. When the value is greater than 0, the distribution is steeper than normal distribution. Negative value implies the reverse.

$$\mathrm{ExcessKurt}[X] = \mathrm{E}\left[\left(\frac{X-\mu}{\sigma}\right)^4\right] - 3 = \sum_{b=0}^{B-1}\left(\frac{x_b-\mu}{\sigma}\right)^4 p(b) - 3$$

, where μ and σ are the mean and standard-deviation of the VOI, respectively.

Texture_Histo_Energy

Energy

Simpler images produce bigger values.

$$\mathrm{Energy} = \sum_{b=0}^{B-1} p(b)^2$$

Texture_Histo_Entropy

Entropy

It measures the uncertainty or randomness in the image values.

$$\mathrm{Entropy} = -\sum_{b=0}^{B-1} p(b)\log_2 p(b)$$

Texture_Histo_Min

Minimum value

The minimum value inside the VOI. It is noted that the gray-values shall be truncated to 'MinPixelValue' so that the minimum value cannot be lower than 'MinPixelValue'

Texture_Histo_Max

Maximum value

The maximum value inside the VOI. It is noted that the gray-values shall be truncated to 'MaxPixelValue' so that the maximum value cannot exceed 'MaxPixelValue'

Texture_Histo_VoxelCount

Number of voxels in VOI

It measures the size of VOI in number of voxels.

2.5 Percentile Values

A percentile is a measure used in statistics indicating the value below a given percentage of gray-values in VOI. For example, the 25th percentile is the gray value below which 25% of the gray-values may be found.

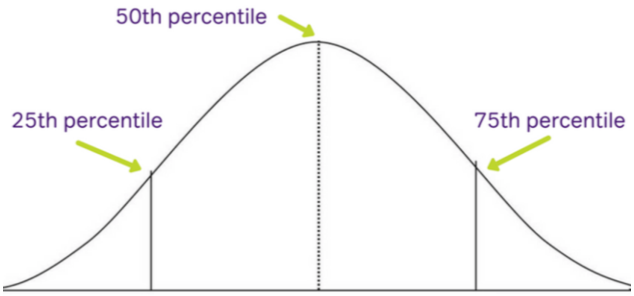

Texture_Percentile_10

The 10th percentile value.

Texture_Percentile_25

The 25th percentile value.

Texture_Percentile_50

The 50th percentile value, which is the median value.

Texture_Percentile_75

The 75th percentile value.

Texture_Percentile_90

The 90th percentile value.

Texture_Percentile_95

The 95th percentile value.

2.6 Gradient Features

Gradient image is the filtered image to produce the difference values from the neighboring voxels, so the gradient value shall be higher if there is a big edge or complex patterned texture. The following figures show an example. The right figure is the gradient image of the left figure.

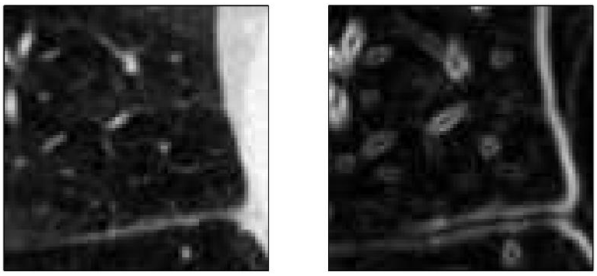

Texture_Grad_Mean

Mean of gradient values

The average value of the magnitude of the gradient images. The high value implies the VOI has big edges or complex patterned texture.

Texture_Grad_Std

Standard deviation of gradient values

The standard deviation value of gradient image.

2.7 GLCM Features (Haralick Features)

GLCM is a short term for 'Gray-Level Cooccurrence Matrices.' For the detailed information, refer to the following technical publication: Robert M. Haralick, K. Shanmugam, Its'hak Dinstein, "Textural Features for Image Classification," *IEEE Trans. On Systems, Man, and Cybernetics.* SMC-3 (6): 610-621. 1973.

The parameter 'GLCMBins' is designated as 'N_g' for simplicity in this chapter. The GLCM matrix of size $N_g \times N_g$ describes the second-order joint probability function of an image VOI constrained by the mask. The $[i, j]$-th element of the matrix, $P(i, j)$, is the number of times the combination of levels i and j occur in two adjacent voxels in the image. $p(i, j)$ is defined as the normalized value of $P(i, j)$.

As for the adjacency, 4 directions are considered in 2-dimensional images as shown in the following figure, while 13 directions are considered in 3-dimensional images.

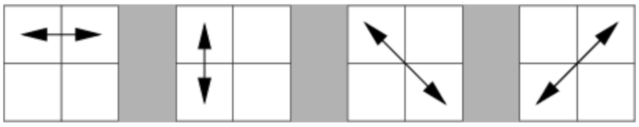

So, there are 4 GLCM in 2D images and 13 GLCM in 3D images according to the directions. The value of the feature is calculated on the GLCM for each direction separately and then finally the mean of these values is used as the final feature value.

From the GLCM, various features are calculated as explained in the followings. For the rest of the document, the following values are defined to calculate GLCM features.

$$p_x(i) = \sum_{j=0}^{N_g-1} p(i,j)$$

$$p_y(j) = \sum_{i=0}^{N_g-1} p(i,j)$$

$$p_{x+y}(k) = \sum_{i=0}^{N_g-1} \sum_{j=0}^{N_g-1} p(i,j)\text{, where } i+j=k\text{, and } k=0,1,\dots,2N_g-2$$

$$p_{x-y}(k) = \sum_{i=0}^{N_g-1} \sum_{j=0}^{N_g-1} p(i,j)\text{, where } |i-j|=k\text{, and } k=0,1,\dots,N_g-1$$

$$\mu_x = \sum_{i=0}^{N_g-1}\sum_{j=0}^{N_g-1}(i+1)p(i,j) = \sum_{i=0}^{N_g-1}(i+1)p_x(i),$$

$$\mu_y = \sum_{i=0}^{N_g-1}\sum_{j=0}^{N_g-1}(j+1)p(i,j) = \sum_{j=0}^{N_g-1}(j+1)p_y(j),$$

$$\sigma_x = \sqrt{\sum_{i=0}^{N_g-1}\sum_{j=0}^{N_g-1}(i+1-\mu_x)^2 p(i,j)} = \sqrt{\sum_{i=0}^{N_g-1}(i+1-\mu_x)^2 p_x(i)} = \sqrt{\sum_{i=0}^{N_g-1}(i+1)^2 p_x(i) - \mu_x{}^2},$$

$$\sigma_y = \sqrt{\sum_{i=0}^{N_g-1}\sum_{j=0}^{N_g-1}\left(j+1-\mu_y\right)^2 p(i,j)} = \sqrt{\sum_{j=0}^{N_g-1}\left(j+1-\mu_y\right)^2 p_y(j)} = \sqrt{\sum_{j=0}^{N_g-1}(j+1)^2 p_y(j) - \mu_y{}^2}$$

$$HX = -\sum_{i=0}^{N_g-1} p_x(i)\log_2 p_x(i)$$

$$HY = -\sum_{j=0}^{N_g-1} p_y(j)\log_2 p_y(j)$$

$$HXY = -\sum_{i=0}^{N_g-1}\sum_{j=0}^{N_g-1} p(i,j)\log_2 p(i,j)$$

$$HXY1 = -\sum_{i=0}^{N_g-1}\sum_{j=0}^{N_g-1} p(i,j)\log_2\left(p_x(i)p_y(j)\right)$$

$$HXY2 = -\sum_{i=0}^{N_g-1}\sum_{j=0}^{N_g-1} p_x(i)p_y(j)\log_2\left(p_x(i)p_y(j)\right)$$

Texture_GLCM_ASM

Angular Second Momentum of GLCM

It is called as 'Joint Energy'. ASM for a single direction is calculated by;

$$ASM = \sum_{i=0}^{N_g-1}\sum_{j=0}^{N_g-1} p(i,j)^2$$

Texture_GLCM_IDM

Inverse Difference Moment of GLCM

$$IDM = \sum_{k=0}^{N_g-1} \frac{p_{x-y}(k)}{1+k^2}$$

Texture_GLCM_IDMN

Inverse Difference Moment Normalized

$$IDMN = \sum_{k=0}^{N_g-1} \frac{p_{x-y}(k)}{1+\left(\frac{k}{N_g}\right)^2}$$

Texture_GLCM_Homogeneity

Homogeneity value of GLCM. It is called as 'Inverse Difference (ID)'

$$\text{Homogeneity} = \sum_{k=0}^{N_g-1} \frac{p_{x-y}(k)}{1+k}$$

Texture_GLCM_HomogeneityNormalized

Normalized homogeneity value of GLCM. It is also known as 'Inverse Differece Normalized (IDN)'.

$$IDN = \sum_{k=0}^{N_g-1} \frac{p_{x-y}(k)}{1+\frac{k}{N_g}}$$

Texture_GLCM_InverseVariance

Inverse variance

$$\text{Inverse variance} = \sum_{k=1}^{N_g-1} \frac{p_{x-y}(k)}{k^2}$$

Note that $k = 0$ case is excluded in the calculation.

Texture_GLCM_Contrast

Contrast value of GLCM

It measures the local intensity variation.

$$\text{Contrast} = \sum_{k=0}^{N_g-1} k^2 p_{x-y}(k)$$

Texture_GLCM_Correlation

Correlation value of GLCM

$$\text{Correlation} = \frac{\sum_{i=0}^{N_g-1}\sum_{j=0}^{N_g-1}(i+1-\mu_x)(j+1-\mu_y)p(i,j)}{\sigma_x\sigma_y} = \frac{\sum_{i=0}^{N_g-1}\sum_{j=0}^{N_g-1}(i+1)(j+1)p(i,j)-\mu_x\mu_y}{\sigma_x\sigma_y}$$

Texture_GLCM_Autocor

Autocorrelation

$$\text{Autocor} = \sum_{i=0}^{N_g-1}\sum_{j=0}^{N_g-1}(i+1)(j+1)p(i,j)$$

Texture_GLCM_Entropy

Entropy value of GLCM

$$\text{Entropy} = -\sum_{i=0}^{N_g-1}\sum_{j=0}^{N_g-1}p(i,j)\log_2(p(i,j))$$

Texture_GLCM_CP

Cluster Prominence

It implies the skewness and asymmetry of the GLCM.

$$CP = \sum_{i=0}^{N_g-1} \sum_{j=0}^{N_g-1} \left(i + 1 + j + 1 - \mu_x - \mu_y\right)^4 p(i,j)$$

Texture_GLCM_CS

Cluster Shade

It measures the skewness and the uniformity of the GLCM.

$$CS = \sum_{i=0}^{N_g-1} \sum_{j=0}^{N_g-1} \left(i + 1 + j + 1 - \mu_x - \mu_y\right)^3 p(i,j)$$

Texture_GLCM_CT

Cluster Tendency

It measures the groupingness of voxels with similar gray-level values.

$$CT = \sum_{i=0}^{N_g-1} \sum_{j=0}^{N_g-1} \left(i + 1 + j + 1 - \mu_x - \mu_y\right)^2 p(i,j)$$

Texture_GLCM_SumEntropy

Sum Entropy

$$\text{Sum entropy} = -\sum_{k=0}^{2N_g-2} p_{x+y}(k) \cdot \log_2\left(p_{x+y}(k)\right)$$

Texture_GLCM_DiffAverage

Difference Average

$$DiffAverage = \sum_{k=0}^{N_g-1} k \cdot p_{x-y}(k)$$

Texture_GLCM_DiffEntropy

Difference Entropy

$$DiffEntropy = -\sum_{k=0}^{N_g-1} p_{x-y}(k) \cdot \log_2\left(p_{x-y}(k)\right)$$

Texture_GLCM_DiffVariance

Difference variance

Difference Variance is a measure of heterogeneity that places higher weights on differing intensity level pairs that deviate more from the mean.

$$\text{Difference variance} = \sum_{k=0}^{N_g-1} (k - DA)^2 p_{x-y}(k)$$

, where DA is the difference average.

Texture_GLCM_IMC1

Informational measure of correlation 1

$$IMC1 = \frac{HXY - HXY1}{\max\{HX, HY\}}$$

When both HX and HY are zero, the value is regarded as zero.

Texture_GLCM_IMC2

Informational measure of correlation 2

$$IMC2 = \sqrt{1 - e^{-2(HXY2 - HXY)}}$$

Sometimes floating-point precision may cause a case of $HXY2 < HXY$. In this case, the value is regarded as zero.

Texture_GLCM_MCC

Maximal correlation coefficient

It is a measure of complexity of the texture. The value should be between 0 and 1. In case of flat region, the value becomes 1.

$$MCC = \sqrt{\text{second largest eigenvalue of Q}}$$

$$Q(i,j) = \sum_{k=0}^{N_g-1} \frac{p(i,k)p(j,k)}{p_x(i)p_y(j)}$$

Texture_GLCM_MaxProb

Maximum probability

The maximum value of the normalized GLCM elements.

$$\text{Maximum probability} = \max\left(p(i,j)\right)$$

Texture_GLCM_SumAverage

Sum average

Sum Average measures the relationship between occurrences of pairs with lower intensity values and occurrences of pairs with higher intensity values.

$$\text{Sum average} = \sum_{k=0}^{2N_g-2} p_{x+y}(k)(k+2)$$

2.8 GLRLM Features

GLRLM is a short term for 'Gray Level Run Length Matrix.' It quantifies gray level runs, which are defined as the length of consecutive voxels that have the same gray level value.

The $[i, j]$-th element of the matrix, $P(i, j)$, is the number of runs with gray level i and length j occur in the VOI. Similarly to GLCM calculation, the GLRLM matrix is calculated along 13 directions in 3D images. The value of the feature is calculated on the GLCM for each direction separately and then finally the mean of these values is used as the final feature value.

(Example)

For an image $\mathbf{I}$, $I = \begin{pmatrix} 5 & 2 & 5 & 4 & 4 \\ 3 & 3 & 3 & 1 & 3 \\ 2 & 1 & 1 & 1 & 3 \\ 4 & 2 & 2 & 2 & 3 \\ 3 & 5 & 3 & 3 & 2 \end{pmatrix}$

The GLRLM for x-direction becomes:

$$P = \begin{pmatrix} 1 & 0 & 1 & 0 & 0 \\ 3 & 0 & 1 & 0 & 0 \\ 4 & 1 & 1 & 0 & 0 \\ 1 & 1 & 0 & 0 & 0 \\ 3 & 0 & 0 & 0 & 0 \end{pmatrix}$$

Let:

G be the number of intensity values in the image

L be the number of run length in the image

Nr be the number of runs in the image for a direction, which is equal to $\sum_{g=0}^{G-1}\sum_{l=0}^{L-1} P(g,l)$

$p(g,l)$ be the normalized run length matrix, defined as $p(g,l) = \frac{P(g,l)}{Nr}$

Texture_GLRLM_SRE

Short Run Emphasis

It is a measure of the distribution of short run lengths, with a greater value indicative of shorter run lengths and more fine textural textures.

$$SRE = \sum_{g=0}^{G-1}\sum_{l=0}^{L-1} \frac{p(g,l)}{(l+1)^2}$$

Texture_GLRLM_LRE

Long Runs Emphasis

It is a measure of the distribution of long run lengths, with a greater value indicative of longer run lengths and more coarse structural textures.

$$LRE = \sum_{g=0}^{G-1}\sum_{l=0}^{L-1} p(g,l)\cdot(l+1)^2$$

Texture_GLRLM_LGRE

Low Gray-level Run Emphasis

It measures the distribution of low gray-level values, with a higher value indicating a greater concentration of low gray-level values in the image.

$$LGRE = \sum_{g=0}^{G-1}\sum_{l=0}^{L-1} \frac{p(g,l)}{(g+1)^2}$$

Texture_GLRLM_HGRE

High Gray-level Run Emphasis

It measures the distribution of high gray-level values, with a higher value indicating a greater concentration of high gray-level values in the image.

$$HGRE = \sum_{g=0}^{G-1}\sum_{l=0}^{L-1} p(g,l)\cdot(g+1)^2$$

Texture_GLRLM_SRLGE

Short-Run Low Gray-level Emphasis

It measures the joint distribution of shorter run lengths with lower gray-level values.

$$SRLGE = \sum_{g=0}^{G-1}\sum_{l=0}^{L-1} \frac{p(g,l)}{(g+1)^2\cdot(l+1)^2}$$

Texture_GLRLM_SRHGE

Short-Run High Gray-level Emphasis

It measures the joint distribution of shorter run lengths with higher gray-level values.

$$SRHGE = \sum_{g=0}^{G-1}\sum_{l=0}^{L-1}\frac{p(g,l)}{(l+1)^2}(g+1)^2$$

Texture_GLRLM_LRLGE

Long-Run Low Gray-level Emphasis

It measures the joint distribution of long run lengths with lower gray-level values.

$$LRLGE = \sum_{g=0}^{G-1}\sum_{l=0}^{L-1}\frac{p(g,l)}{(g+1)^2}(l+1)^2$$

Texture_GLRLM_LRHGE

Long-Run High Gray-level Emphasis

It measures the joint distribution of long run lengths with higher gray-level values.

$$LRHGE = \sum_{g=0}^{G-1}\sum_{l=0}^{L-1}p(g,l)\cdot(g+1)^2\cdot(l+1)^2$$

Texture_GLRLM_GNUN

Gray-level Non-Uniformity Normalized

If gray-levels are similar, the value would be lower.

$$GNUN = \sum_{g=0}^{G-1}\left(\sum_{l=0}^{L-1}p(g,l)\right)^2$$

Texture_GLRLM_RLNUN

Run-Length Non-Uniformity Normalized

It measures the similarity of run lengths throughout the image, with a lower value indicating more homogeneity among run lengths in the image.

$$RLNUN = \sum_{l=0}^{L-1}\left(\sum_{g=0}^{G-1} p(g,l)\right)^2$$

Texture_GLRLM_RP

Run Percentage

RP measures the coarseness of the texture by taking the ratio of number of runs and number of voxels in the VOI.

$$RP = \frac{N_r}{N_p}$$

, where N_p is the total number of voxels.

Texture_GLRLM_RV

Run Variance

It is the variance in runs for the run lengths.

$$RV = \sum_{g=0}^{G-1}\sum_{l=0}^{L-1} p(g,l)\cdot(l+1-\mu_l)^2\text{, where } \mu_l = \sum_{g=0}^{G-1}\sum_{l=0}^{L-1} p(g,l)\cdot(l+1)$$

Texture_GLRLM_RE

Run Entropy

It measures the uncertainty or the randomness in the distribution of run lengths and gray levels. A higher value indicates more heterogeneity in the texture patterns.

$$RE = -\sum_{g=0}^{G-1}\sum_{l=0}^{L-1} p(g,l)\log_2(p(g,l))$$

Texture_GLRLM_GLV

Gray Level Variance

It is the variance in gray-level in the image.

$$GLV = \sum_{g=0}^{G-1}\sum_{l=0}^{L-1} p(g,l)(g+1-\mu_g)^2\text{, where } \mu_g = \sum_{g=0}^{G-1}\sum_{l=0}^{L-1} p(g,l)\cdot(g+1)$$

2.9 GLSZM Features

GLSZM is a short term for 'Gray Level Size Zone Matrix.' It quantifies gray level zones in an image. A gray level zone is defined as the number of connected voxels that share the same gray level intensity. A voxel is considered connected if the distance is 1 according to the infinity norm. We use 26-connectivity in 3D VOI. The (g,s)-th element of the matrix, $P(g,s)$, is the number of zones with gray-level g and size s. Contrary to GLCM and GLRLM, the GLSZM is rotation-invariant, with only one matrix calculated for all directions in the VOI.

Please refer to the technical publication for detail: Guillaume Thibault; Bernard Fertil; Claire Navarro; Sandrine Pereira; Pierre Cau; Nicolas Levy; Jean Sequeira; Jean-Luc Mari (2009). "Texture Indexes and Gray Level Size Zone Matrix. Application to Cell Nuclei Classification". *Pattern Recognition and Information Processing* (PRIP): 140-145.

The distance is set to 1 by default.

(Example)

Texture

1	2	3	4
1	3	4	4
3	2	2	2
4	1	4	1

$\Longrightarrow$

Gray level g_m	*Size zone* (s_n) 1	2	3
1	2	1	0
2	1	0	1
3	0	0	1
4	2	0	1

Texture

1	1	3	4
1	3	4	4
3	2	4	4
3	2	1	1

$\Longrightarrow$

Gray level g_m	*Size zone* (s_n) 1	2	3	4	5
1	0	1	1	0	0
2	0	1	0	0	0
3	0	0	0	1	0
4	0	0	0	0	1

G The number of gray-levels

S The number of zones

N The number of voxels inside the VOI

Z The number of zones inside the VOI.

$$Z = \sum_{g=1}^{G}\sum_{s=1}^{S} P(i,j) \text{ and } 1 \leq Z \leq N$$

$P(g,s)$ The element of the GLSZM

$p(g,s)$ The element of the normalized GLSZM. $p(g,s) = \frac{P(g,s)}{Z}$

Texture_GLSZM_SAE

Small Area Emphasis

It is a measure of the distribution of small size zones, with a greater value indicating smaller size zones and more fine textures.

$$SAE = \frac{\sum_{g=0}^{G-1}\sum_{s=0}^{S-1}\frac{P(g,s)}{(s+1)^2}}{Z} = \sum_{g=0}^{G-1}\sum_{s=0}^{S-1}\frac{p(g,s)}{(s+1)^2}$$

Texture_GLSZM_LAE

Large Area Emphasis

It is a measure of the distribution of large area size zones, with a greater value indicating larger size zones and more coarse textures.

$$LAE = \frac{\sum_{g=0}^{G-1}\sum_{s=0}^{S-1}P(g,s)(s+1)^2}{Z} = \sum_{g=0}^{G-1}\sum_{s=0}^{S-1}p(g,s)(s+1)^2$$

Texture_GLSZM_GLN

Gray Level Nonuniformity

It measures the variability of grayl-leve intensity values in the image, with a lower value indicating a greater similarity in intensity values.

$$GLN = \frac{\sum_{g=0}^{G-1}\left(\sum_{s=0}^{S-1}P(g,s)\right)^2}{Z} = Z\sum_{g=0}^{G-1}\left(\sum_{s=0}^{S-1}p(g,s)\right)^2$$

Texture_GLSZM_GLNN

Gray Level Nonuniformity Normalized

It is the normalized version of Texture_GLSZM_GLN

$$GLNN = \frac{\sum_{g=0}^{G-1}\left(\sum_{s=0}^{S-1}P(g,s)\right)^2}{Z^2} = \sum_{g=0}^{G-1}\left(\sum_{s=0}^{S-1}p(g,s)\right)^2$$

Texture_GLSZM_SZN

Size Zone Nonuniformity

It measures the variability of size zone volumes throughout the image, with a lower value indicating more homogeneity among zone size volume in the image.

$$SZN = \frac{\sum_{s=0}^{S-1}\left(\sum_{g=0}^{G-1} P(g,s)\right)^2}{Z} = Z\sum_{s=0}^{S-1}\left(\sum_{g=0}^{G-1} p(g,s)\right)^2$$

Texture_GLSZM_SZNN

Size Zone Nonuniformity Normalized

It is the normalized version of Texture_GLSZM_SZN

$$SZNN = \frac{\sum_{s=0}^{S-1}\left(\sum_{g=0}^{G-1} P(g,s)\right)^2}{Z^2} = \sum_{s=0}^{S-1}\left(\sum_{g=0}^{G-1} p(g,s)\right)^2$$

Texture_GLSZM_ZP

Zone Percentage

It measures the coarseness of the texture by taking the ratio of number of zones and number of voxels in the VOI. Values are in range $\frac{1}{N} \leq ZP \leq 1$, with higher values indicating a larger portion of the VOI consists of small zones.

$$ZP = \frac{Z}{N}$$

Texture_GLSZM_GLV

Gray Level Variance

It is the variance in gray level intensities for the zones.

$$GLV = \sum_{g=0}^{G-1}\sum_{s=0}^{S-1} p(g,s)(g+1-\mu_g)^2 \text{, where } \mu_g = \sum_{g=0}^{G-1}\sum_{s=0}^{S-1} p(g,s)(g+1)$$

Texture_GLSZM_ZV

Zone Variance

It is the variance in zone size volumes for the zones.

$$ZV = \sum_{g=0}^{G-1}\sum_{s=0}^{S-1} p(g,s)(s+1-\mu_s)^2 \text{ , where } \mu_s = \sum_{g=0}^{G-1}\sum_{s=0}^{S-1} p(g,s)(s+1)$$

Texture_GLSZM_ZE

Zone Entropy

It measures the uncertainty or the randomness in the distribution of zone sizes and gray levels. A higher value indiciates more heterogeneity in the texture patterns.

$$ZE = -\sum_{g=0}^{G-1}\sum_{s=0}^{S-1} p(g,s)\log_2 p(g,s)$$

Texture_GLSZM_LGLZE

Low Gray Level Zone Emphasis

It measures the distribution of lower gray-level size zones, with a higher value indicating a greater proportion of lower gray-level values and size zones in the image.

$$LGLZE = \frac{\sum_{g=0}^{G-1}\sum_{s=0}^{S-1}\frac{P(g,s)}{(g+1)^2}}{Z} = \sum_{g=0}^{G-1}\sum_{s=0}^{S-1}\frac{p(g,s)}{(g+1)^2}$$

Texture_GLSZM_HGLZE

High Gray Level Zone Emphasis

It measures the distribution of the higher gray-level values, with a higher value indicating a greater proportion of higher gray-level values and size zones in the image.

$$HGLZE = \frac{\sum_{g=0}^{G-1}\sum_{s=0}^{S-1} P(g,s)(g+1)^2}{Z} = \sum_{g=0}^{G-1}\sum_{s=0}^{S-1} p(g,s)(g+1)^2$$

Texture_GLSZM_SALGLE

Small Area Low Gray Level Emphasis

It measures the proportion in the image of the joint distribution of smaller size zones with lower gray-level values.

$$SALGLE = \frac{\sum_{g=0}^{G-1}\sum_{s=0}^{S-1}\frac{P(g,s)}{(g+1)^2(s+1)^2}}{Z} = \sum_{g=0}^{G-1}\sum_{s=0}^{S-1}\frac{p(g,s)}{(g+1)^2(s+1)^2}$$

Texture_GLSZM_SAHGLE

Small Area High Gray Level Emphasis

It measures the proportion in the image of the joint distribution of smaller size zones with higher gray-level values.

$$SAHGLE = \frac{\sum_{g=0}^{G-1}\sum_{s=0}^{S-1}\frac{P(g,s)(g+1)^2}{(s+1)^2}}{Z} = \sum_{g=0}^{G-1}\sum_{s=0}^{S-1}\frac{p(g,s)(g+1)^2}{(s+1)^2}$$

Texture_GLSZM_LALGLE

Large Area Low Gray Level Emphasis

It measures the proportion in the image of the joint distribution of larger size zones with lower gray-level values.

$$LALGLE = \frac{\sum_{g=0}^{G-1}\sum_{s=0}^{S-1}\frac{P(g,s)(s+1)^2}{(g+1)^2}}{Z} = \sum_{g=0}^{G-1}\sum_{s=0}^{S-1}\frac{p(g,s)(s+1)^2}{(g+1)^2}$$

Texture_GLSZM_LAHGLE

Large Area High Gray Level Emphasis

It measures the proportion in the image of the joint distribution of larger size zones with higher gray-level values.

$$LAHGLE = \frac{\sum_{g=0}^{G-1}\sum_{s=0}^{S-1} P(g,s)(g+1)^2(s+1)^2}{Z} = \sum_{g=0}^{G-1}\sum_{s=0}^{S-1} p(g,s)(g+1)^2(s+1)^2$$

2.10 NGTDM Features

NGTDM is a short term for 'Neighbouring Gray Tone Difference Matrix.' It quantifies the difference between a gray value and the average gray value of its neighbors within distance δ. The sum of absolute differences for gray level i is stored in the matrix. The distance δ is set to 1 by default.

For detail, please refer to the technical publication: Amadasun M, King R; Textural features corresponding to textural properties; *Systems, Man and Cybernetics, IEEE Transactions on 19*:1264-1274 (1989). doi: 10.1109/21.44046

(Example)

For an image **I**, $\mathrm{I} = \begin{bmatrix} 1 & 2 & 5 & 2 \\ 3 & 5 & 1 & 3 \\ 1 & 3 & 5 & 5 \\ 3 & 1 & 1 & 1 \end{bmatrix}$,

NGTDM is calculated as follows.

i	n_i	p_i	s_i
1	6	0.375	13.35
2	2	0.125	2.00
3	4	0.25	2.63
4	0	0.00	0.00
5	4	0.25	10.075

Here, n_i is the number of gray level, i and p_i is n_i divided by total number $N_{v,p}$.

s_i values can be calculated as follows:

$$s_1 = \left|1 - \frac{2+3+5}{3}\right| + \left|1 - \frac{2+5+2+5+3+3+5+5}{8}\right| + \left|1 - \frac{3+5+3+3+1}{5}\right| + \left|1 - \frac{1+3+5+3+1}{5}\right| + \left|1 - \frac{3+5+5+1+1}{5}\right| + \left|1 - \frac{5+5+1}{3}\right| = 13.35$$

$$s_2 = \left|2 - \frac{1+5+3+5+1}{5}\right| + \left|2 - \frac{5+1+3}{3}\right| = 2$$

$$s_3 = \left|3 - \frac{1+2+5+1+3}{5}\right| + \left|3 - \frac{5+2+1+5+5}{5}\right| + \left|3 - \frac{3+5+1+1+5+3+1+1}{8}\right| + \left|3 - \frac{1+3+1}{3}\right| = 3.03$$

$$s_5 = \left|5 - \frac{2+2+5+1+3}{5}\right| + \left|5 - \frac{1+2+5+3+1+1+3+5}{8}\right| + \left|5 - \frac{5+1+3+3+5+1+1+1}{8}\right| + \left|5 - \frac{1+3+5+1+1}{5}\right| = 10.075$$

$N_{v,p}$ The total number of voxels who have neighbours, $\sum n_i$. Note that some voxels have no neighbours.

N_g The number of gray-levels

$N_{g,p}$ The number of gray levels, where $p_i \neq 0$.

Texture_NGTDM_Coarseness

Coarseness is a measure of average difference between the center voxel and its neighbours. It is indicating the spatial rate of change. A higher value implies a lower spatial change rate and a locally more uniform texture.

$$\text{Coarseness} = \frac{1}{\sum_{i=0}^{N_g-1} p_i s_i}$$

If an image is perfectly flat, an arbitrary value of 10^6 would be used.

Texture_NGTDM_Contrast

Contrast is a measure of the spatial intensity change, but is also dependent on the overall gray level dynamic range. The value is high when both the dynamic range and the spatial change rate are high, i.e. an image with a large range of gray levels, with large changes between voxels and their neighbourhood.

$$\text{Contrast} = \left(\frac{1}{N_{g,p}(N_{g,p}-1)} \sum_{i=0}^{N_g-1} \sum_{j=0}^{N_g-1} p_i p_j (i-j)^2\right)\left(\frac{1}{N_{v,p}} \sum_{i=0}^{N_g-1} s_i\right), where\ p_i \neq 0, p_j \neq 0$$

If $N_{g,p}$ is 1, an arbitrary value of 0 would be used.

Texture_NGTDM_Busyness

It measures the change from a pixel to its neighbour. A high value for busyness indicates a 'busy' image, with rapid changes of intensity between pixels and its neighborhood.

$$\text{Busyness} = \frac{\sum_{i=0}^{N_g-1} p_i s_i}{\sum_{i=0}^{N_g-1} \sum_{j=0}^{N_g-1} \left|(i+1)p_i - (j+1)p_j\right|}, where\ p_i \neq 0, p_j \neq 0$$

If $N_{g,p} = 1$, an arbitrary value of 0 would be used.

Texture_NGTDM_Complexity

An image is considered complex when there are many primitive components in the image, i.e. the image is non-uniform and there are many rapid changes in gray level intensity.

$$\text{Complexity} = \frac{1}{N_{v,p}} \sum_{i=0}^{N_g-1} \sum_{j=0}^{N_g-1} |i-j| \frac{p_i s_i + p_j s_j}{p_i + p_j}, where\ p_i \neq 0, p_j \neq 0$$

Texture_NGTDM_Strength

Strength is a measure of the primitives in an image. Its value is high when the primitives are easily defined and visible, i.e. an image with slow change in intensity but more large coarse differences in gray level intensities.

$$\text{Strength} = \frac{\sum_{i=0}^{N_g-1} \sum_{j=0}^{N_g-1} (p_i + p_j)(i-j)^2}{\sum_{i=0}^{N_g-1} s_i}, where\ p_i \neq 0, p_j \neq 0$$

If $\sum_{i=0}^{N_g-1} s_i = 0$, an arbitrary value of 0 would be used.

2.11 GLDM Features

GLDM is a short term for 'Gray Level Dependence Matrix.' It quantifies gray level dependencies in an image. A gray level dependency is defined as the number of connected voxels within distance δ that are dependent on the center voxel. A neighboring voxel with gray level f is considered dependent on center voxel with gray level g if $|g - f| \leq \alpha$. The $[g, d]$-th element of the GLDM, $P(g, d)$, describes the number of times a voxel with gray level g with d dependent voxels in its neighborhood appears in an image.

The distance δ is set to 1, and the cutoff α is set to 0 by default.

(Example)

$$I = \begin{bmatrix} 5 & 2 & 5 & 4 & 4 \\ 3 & 3 & 3 & 1 & 3 \\ 2 & 1 & 1 & 1 & 3 \\ 4 & 2 & 2 & 2 & 3 \\ 3 & 5 & 3 & 3 & 2 \end{bmatrix}$$

When $\delta = 1$, $\alpha = 0$,

$$P = \begin{bmatrix} 0 & 1 & 2 & 1 \\ 1 & 2 & 3 & 0 \\ 1 & 4 & 4 & 0 \\ 1 & 2 & 0 & 0 \\ 3 & 0 & 0 & 0 \end{bmatrix}$$

$P(g, d)$ Dependence matrix element

$p(g, d)$ Normalized value of the dependence matrix element. $p(g, d) = \frac{P(g,d)}{N}$

Texture_GLDM_SDE

Small Dependence Emphasis

A measure of the distribution of small dependencies, with a greater value indicative of smaller dependence and less homogeneous textures.

$$SDE = \frac{\sum_{g=0}^{G-1} \sum_{d=0}^{D-1} \frac{P(g,d)}{(d+1)^2}}{N} = \sum_{g=0}^{G-1} \sum_{d=0}^{D-1} \frac{p(g,d)}{(d+1)^2}$$

Texture_GLDM_LDE

Large Dependence Emphasis

A measure of the distribution of large dependencies, with a greater value indicative of larger dependence and more homogeneous textures.

$$LDE = \frac{\sum_{g=0}^{G-1}\sum_{d=0}^{D-1} P(g,d)(d+1)^2}{N} = \sum_{g=0}^{G-1}\sum_{d=0}^{D-1} p(g,d)(d+1)^2$$

Texture_GLDM_GLN

Gray Level Nonuniformity

Measures the similarity of gray-level intensity values in the image, where a lower value correlates with a greater similarity in intensity values.

$$GLN = \frac{\sum_{g=0}^{G-1}\left(\sum_{d=0}^{D-1} P(g,d)\right)^2}{N} = N\sum_{g=0}^{G-1}\left(\sum_{d=0}^{D-1} p(g,d)\right)^2$$

Texture_GLDM_DN

Dependence Nonuniformity

Measures the similarity of dependence throughout the image, with a lower value indicating more homogeneity among dependencies in the image.

$$DN = \frac{\sum_{d=0}^{D-1}\left(\sum_{g=0}^{G-1} P(g,d)\right)^2}{N} = N\sum_{d=0}^{D-1}\left(\sum_{g=0}^{G-1} p(g,d)\right)^2$$

Texture_GLDM_DNN

Dependence Nonuniformity Normalized

It is the normalized version of Texture_GLDM_DN.

$$DNN = \frac{\sum_{d=0}^{D-1}\left(\sum_{g=0}^{G-1} P(g,d)\right)^2}{N^2} = \sum_{d=0}^{D-1}\left(\sum_{g=0}^{G-1} p(g,d)\right)^2$$

Texture_GLDM_GLV

Gray Level Variance

The variance in gray level in the image.

$$GLV = \sum_{g=0}^{G-1}\sum_{d=0}^{D-1} p(g,d)\left(g+1-\mu_g\right)^2, \qquad where\ \mu_g = \sum_{g=0}^{G-1}\sum_{d=0}^{D-1}(g+1)\cdot p(g,d)$$

Texture_GLDM_DV

Dependence Variance

The variance in dependence size in the image

$$DV = \sum_{g=0}^{G-1}\sum_{d=0}^{D-1} p(g,d)(d+1-\mu_d)^2, \qquad where\ \mu_d = \sum_{g=0}^{G-1}\sum_{d=0}^{D-1}(d+1)\cdot p(g,d)$$

Texture_GLDM_DE

Dependence Entropy

The uncertaintiy/randomness of dependence matrix.

$$\text{Dependence entropy} = -\sum_{g=0}^{G-1}\sum_{d=0}^{D-1} p(g,d)\log_2 p(g,d)$$

Texture_GLDM_LGLE

Low Gray Level Emphasis

Measures the distribution of low gray-level values, with a higher value indicating a greater concentration of low gray-level values in the image.

$$LGLE = \frac{\sum_{g=0}^{G-1}\sum_{d=0}^{D-1}\frac{P(g,d)}{(g+1)^2}}{N} = \sum_{g=0}^{G-1}\sum_{d=0}^{D-1}\frac{p(g,d)}{(g+1)^2}$$

Texture_GLDM_HGLE

High Gray Level Emphasis

Measures the distribution of the higher gray-level values, with a higher value indicating a greater concentration of high gray-level values in the image.

$$HGLE = \frac{\sum_{g=0}^{G-1}\sum_{d=0}^{D-1} P(g,d)(g+1)^2}{N} = \sum_{g=0}^{G-1}\sum_{d=0}^{D-1} p(g,d)(g+1)^2$$

Texture_GLDM_SDLGLE

Small dependence low gray level emphasis

It measures the joint distribution of small dependence with lower gray-level values.

$$SDLGLE = \frac{\sum_{g=0}^{G-1}\sum_{d=0}^{D-1} \frac{P(g,d)}{(g+1)^2(d+1)^2}}{N} = \sum_{g=0}^{G-1}\sum_{d=0}^{D-1} \frac{p(g,d)}{(g+1)^2(d+1)^2}$$

Texture_GLDM_SDHGLE

Small Dependence High Gray Level Emphasis

It measures the joint distribution of small dependence with higher gray-level values.

$$SDHGLE = \frac{\sum_{g=0}^{G-1}\sum_{d=0}^{D-1} \frac{P(g,d)(g+1)^2}{(d+1)^2}}{N} = \sum_{g=0}^{G-1}\sum_{d=0}^{D-1} \frac{p(g,d)(g+1)^2}{(d+1)^2}$$

Texture_GLDM_LDLGLE

Large Dependence Low Gray Level Emphasis

It measures the joint distribution of large dependence with lower gray-level values.

$$LDLGLE = \frac{\sum_{g=0}^{G-1}\sum_{d=0}^{D-1} \frac{P(g,d)(d+1)^2}{(g+1)^2}}{N} = \sum_{g=0}^{G-1}\sum_{d=0}^{D-1} \frac{p(g,d)(d+1)^2}{(g+1)^2}$$

Texture_GLDM_LDHGLE

Large Dependence High Gray Level Emphasis

It measures the joint distribution of large dependence with higher gray-level values.

$$LDHGLE = \frac{\sum_{g=0}^{G-1}\sum_{d=0}^{D-1} P(g,d)(g+1)^2(d+1)^2}{N} = \sum_{g=0}^{G-1}\sum_{d=0}^{D-1} p(g,d)(g+1)^2(d+1)^2$$

2.12 Moment Features

The moment is a well-known as a very robust feature which is invariant to image scale and rotation. For the technical detail, please refer to the following publication: F. A. Sadjadi and E. L. Hall, "Three-dimensional moment invariants," *IEEE Trans. on Pattern Analysis and Machine Intelligence*, 2(2), pp. 127-136, April 1980.

$$m_{\mathrm{ijk}} = \sum_{x,y,z} \mathrm{Image}(x,y,z) \cdot x^i y^j z^k$$

$$\mu_{\mathrm{ijk}} = \sum_{x,y,z} \mathrm{Image}(x,y,z) \cdot (x-\overline{x})^i \cdot (y-\overline{y})^j \cdot (z-\overline{z})^k$$

, where $\overline{x} = \frac{m_{100}}{m_{000}}$, $\overline{y} = \frac{m_{010}}{m_{000}}$ and $\overline{z} = \frac{m_{001}}{m_{000}}$

$$\eta_{ijk} = \frac{\mu_{ijk}}{m_{000}^{\frac{i+j+k}{3}+1}}$$

Texture_Moment_J1

$$J_1 = \eta_{200} + \eta_{020} + \eta_{002}$$

Texture_Moment_J2

$$J_2 = \eta_{200}\eta_{020} + \eta_{200}\eta_{002} + \eta_{020}\eta_{002} - \eta_{110}^2 - \eta_{101}^2 - \eta_{011}^2$$

Texture_Moment_J3

$$J_3 = \eta_{200}\eta_{020}\eta_{002} + 2\eta_{110}\eta_{101}\eta_{011} - \eta_{002}\eta_{110}^2 - \eta_{020}\eta_{101}^2 - \eta_{200}\eta_{011}^2$$

3 Shape Features

3.1 Cautions

Shape feature values are susceptible to segmentation mask shapes.

3.2 Shape Features in 3D-View

Shape3D_Volume(mm3)

The volume of segmentation mask in mm^3.

It counts the number of voxels inside the mask, and multiplies the voxel resolution which are extracted from DICOM header information.

$$\text{Volume } (V) = \text{MaskVoxelCount} \times \text{PixelSpacing}_x \times \text{PixelSpacing}_y \times \text{SliceGap}$$

Shape3D_SurfaceArea(mm2)

The surface area in mm^2 is calculated from the boundary voxels as followings.

$$\text{SurfaceArea } (A) = \text{SurfaceVoxelCount} \times \left(\text{PixelSpacing}_x \times \text{PixelSpacing}_y \times \text{SliceGap}\right)^{2/3}$$

Shape3D_SurfaceAreaToVolumeRatio

The ratio of Surface area to volume.

$$\text{Surface to volume ratio } = \frac{A}{V}$$

Shape3D_Sphericity

Sphericity indicates how similar the mask shape is to sphere.

$$\text{Sphericity} = \frac{\pi^{1/3}(6 \cdot V)^{2/3}}{A}$$

Shape3D_Compactness

Compactness of the mask shape.

$$C_D = \frac{A_C - A_{Cmin}}{A_{Cmax} - A_{Cmin}}$$

$$A_C = \frac{6N - A}{2}$$
$$A_{Cmin} = (N - 1)$$
$$A_{Cmax} = 3\left(N - \sqrt[3]{N^2}\right)$$

, where N is the voxel count and A is the surface area.

Refer to the following technical publication for detail: R. S. Montero and E. Bribiesca, "State of the Art of Compactness and Circularity Measures," *International Mathematical Forum*, 4, 2009, no.27, 1305-1335

Shape3D_Compactness2

It also measures the compactness of the shape mask in another way.

$$\text{Compactness2} = \frac{V}{\sqrt{\pi A^3}}$$

The value is in range $0 < \text{compactness2} \le \frac{1}{6\pi}$. If the shape mask is a perfect sphere, the value shall be $\frac{1}{6\pi}$.

Shape3D_Compactness3

Another measure for the compactness of the shape mask.

$$\text{Compactness3} = 36\pi\frac{V^2}{A^3}$$

The value is in range, $0 < \text{compactness3} \le 1$. A perfect sphere shall have a value of 1. It should be mentioned that $\text{compactness3} = (sphericity)^3$

Shape3D_Roundness

3D roundness is called as angularity, R.

To calculate R, first the surface voxels should be extracted.

$$R = \sqrt{\frac{\hat{R}}{\frac{1}{N}\Sigma_1^N(|\hat{R} - R_n| + \hat{R})}}$$

N Surface voxel count.

R_n the distance between n-th surface voxel and the centroid of the mask.

$$\hat{R} = \frac{1}{N}\sum_1^N R_n$$

Shape3D_Circularity

Another measure for 3D roundness.

$$\text{Circularity} = 6\sqrt{\pi} \times \frac{V}{A^{3/2}}$$

Shape3D_SphericalDisproportion

It is the inverse of Sphericity.

$$\text{Spherical disproportion} = \frac{A}{\sqrt[3]{36\pi V^2}}$$

The value is in range, $\text{spherical disproportion} \geq 1$. A perfect sphere shall have a value of 1.

Shape3D_Longest1stAxis(mm)

It measures the distance of two farthest voxels in 3D space. The line connecting these two voxels is defined as 'the 1st longest axis'.

Shape3D_Longest2ndAxis(mm)

The value is the distance of two farthest pixels in all the 2D planes, which are perpendicular to `the 1st longst axis`.

Shape3D_Longest1stAxisOnAxial(mm)

The value is the distance of the two farthest pixels on all the axial images. The line connecting these two pixels is defined as the `1st longest axis on axial`.

Shape3D_Longest2ndAxisOnAxial(mm)

The value is the distance of two farthest pixels in all the 2D planes, which are perpendicular to `the 1st longst axis on axial`.

Shape3D_Longest1stAxisOnSagittal(mm)

The value is the distance of the two farthest pixels on all the sagittal images. The line connecting these two pixels is defined as the `1st longest axis on sagittal`.

Shape3D_Longest2ndAxisOnSagittal(mm)

The value is the distance of two farthest pixels in all the 2D planes, which are perpendicular to `the 1st longst axis on sagittal`.

Shape3D_Longest1stAxisOnCoronal(mm)

The value is the distance of the two farthest pixels on all the coronal images. The line connecting these two pixels is defined as the `1st longest axis on coronal`.

Shape3D_Longest2ndAxisOnCoronal(mm)

The value is the distance of two farthest pixels in all the 2D planes, which are perpendicular to `the 1st longst axis

on coronal`.

Shape3D_PCA1stMajorStd(mm)

The standard deviation value of the 1st major axis from PCA (Principal Component Analysis).

Shape3D_PCA2ndMajorStd(mm)

The standard deviation value of the 2nd major axis from PCA (Principal Component Analysis).

Shape3D_PCA3rdMajorStd(mm)

The standard deviation value of the 3rd axis from PCA (Principal Component Analysis).

Shape3D_Elongation

Elongation is calculated from the 2 major axes from PCA.

$$\text{Elongation} = \sqrt{\frac{\sigma_2}{\sigma_1}}$$

, where σ_1 and σ_2 are the standard deviation of the 1st major axis and 2nd major axis.

Shape3D_Flatness

Flatness is calculated from the major axis and minor axis from PCA.

$$\text{Flatness} = \sqrt{\frac{\sigma_3}{\sigma_1}}$$

, where σ_1 and σ_3 are the standard deviation of the 1st major axis and 3rd major axis.

3.3 Shape Features in 2D-View

Shape2D_Area(mm2)

Area is calcaulated from the number of mask pixels and pixel spacing information from the DICOM headers.

$$\text{Area } (A) = \text{MaskPixelCount} \times \text{PixelSpacing}_x \times \text{PixelSpacing}_y$$

Shape2D_Perimeter(mm)

The perimeter of the 2D mask is the length of the boundary pixels. If a segmentation mask has inner holes, the perimeter of the inner holes would be included.

Shape2D_PerimeterToAreaRatio

Perimeter to area ratio

$$\text{Perimeter to area ratio} = \frac{\text{Perimeter}}{A}$$

Shape2D_Circularity

Circularity indicates how similar the mask is to a circle.

$$\text{Circularity} = \frac{4\pi \times A}{\text{Perimeter}^2}$$

The value of a perfect circle shall be 1.

Shape2D_Compactness

Compactness of the 2D mask is calculated from the followings.

$$C_{DN} = \frac{C_D - C_{Dmin}}{C_{Dmax} - C_{Dmin}}$$

$$C_D = \frac{4N - P}{2}$$
$$C_{Dmin} = (N - 1)$$
$$C_{Dmax} = 2(N - \sqrt{N})$$

, where N is the pixel count and P is the perimeter.

See the technical publication for detail: R. S. Montero and E. Bribiesca, "State of the Art of Compactness and Circularity Measures," *International Mathematical Forum*, 4, 2009, no.27, 1305-1335

Shape2D_Roundness

2D roundness (so called, angularity), R, is calculated from the boundary pixels, as follows.

$$R = \sqrt{\frac{\hat{R}}{\frac{1}{N}\sum_1^N(|\hat{R} - R_n| + \hat{R})}}$$

, where

N Boundary pixel count.

R_n The distance between the n-th boundary pixel and the centroid of the mask.

$$\hat{R} = \frac{1}{N}\sum_1^N R_n$$

Shape2D_LongestAxis(mm)

It measures the distance of two farthest mask pixels in 2D mask. The line connecting these two pixels is defined as 'the 1st longest axis'.

Shape2D_OrthogonalAxis(mm)

The value is the distance of two farthest mask pixels in all the 2D lines, which are perpendicular to `the 1st longst axis`.

Shape2D_PCAMajorStd(mm)

The standard deviation value of the 1st major axis from PCA (Principal Component Analysis) of the mask pixels.

Shape_PCAMinorStd(mm)

The standard deviation value of the minor axis from PCA (Principal Component Analysis) of the mask pixels.

Shape2D_Flatness

Flatness is defined as follows.

$$\text{Flatness} = \sqrt{\frac{\sigma_2}{\sigma_1}}$$

, where σ_1 and σ_2 are the standard deviation of the major axis and the minor axis

4 Fractal Features

4.1 Cautions

Similar to shape features, Fractal feature is susceptible to the shape of the segmentation mask. The fractal feature is calculated by box-sliding technique.

The box size is fixed to 64×64×64, and the sliding increment is adjusted by the parameter 'BoxCoundingSlide.' If the parameter changes, fractal feature value would be changed. So, you need to fix the parameter before your research begins and should keep it constant through your research.

BoxCountingSlide

The box-sliding gap in voxels. If this is set too high, the precision of the value would be poor. Also, the lower value of this parameter would increase the computational burden.

Default: 4

4.2 Fractal Features

Fractal Dimension

Inside the VOI, a small box, whose size is 64×64×64, is sliding to calculate fractal dimension at each location. Finally, all the calculated values from all the sliding positions are averaged to be a final value.
At each sliding position, a box is decomposed into 64×64×64, 32×32×32, 16×16×16, 8×8×8, 4×4×4, 2×2×2, 1×1×1 sized boxes and count the number of non-zero boxes. These values are plotted on the log-log scaled graph and approximated as a single line. The fractal dimension is defined as the slope of the line.

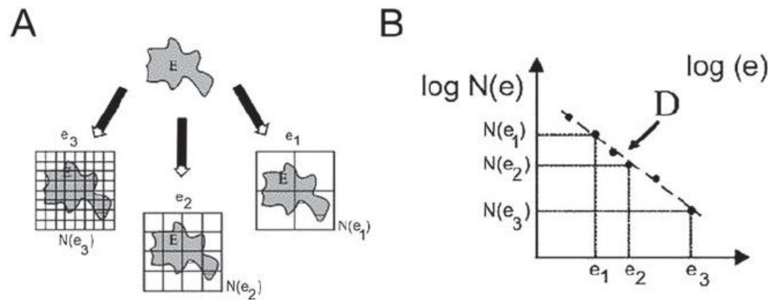

Supplement: S1 File — Republished from PLOS Journal under a CC BY license, with permission from Coreline Soft, original copyright [2021]. (PDF) [file pone.0280523.s001.pdf]
