# Supplementary material for: Radiomics approach to the condylar head for legal age classification using cone-beam computed tomography: A pilot study
Source: PLoS One. 2023 Jan 19;18(1):e0280523. doi: 10.1371/journal.pone.0280523 (PMC9851527; doi:10.1371/journal.pone.0280523)
Supplement: S1 Data — (PDF) [file pone.0280523.s002.pdf]

Patient	Sex	Age	Compactness2	SphericalDisproportion	Longest2ndAxisOnCoronal(mm)	Flatness	FirstOrder_TotalEnergy	FirstOrder_Min	FirstOrder_Max	FirstOrder_RMS
1	F	10	0.022965	1.747509	3.030303	0.565884	15676547784	3985	7485	12983.25941
2	F	10	0.020764	1.86895	3.073833	0.534364	15366042012	3994	7610	12797.86414
3	M	10	0.019236	1.966649	2.073663	0.557374	15037467064	3120	7382	11926.57827
4	M	10	0.020184	1.904544	3.203442	0.608613	25579862545	3452	8212	12323.9974
5	M	10	0.021295	1.837726	3.115417	0.567825	12607347806	1986	6946	11320.88853
6	M	10	0.020865	1.862906	3.17155	0.518224	11422849436	2746	7021	11564.65944
7	M	11	0.023255	1.732969	6.903868	0.671884	35958707799	2079	7184	12063.06215
8	M	11	0.023487	1.721525	6.309893	0.637478	38178666391	2270	7020	11879.32533
9	M	11	0.020977	1.856241	5.199561	0.552942	33764241270	2846	8159	12211.66581
10	M	11	0.019185	1.970134	4.846978	0.576621	37077705870	2793	8425	11938.95144
11	M	11	0.022796	1.756139	5.104042	0.534485	35123298906	2879	7244	12101.73121
12	M	11	0.024203	1.687392	6.216524	0.537183	32552501872	2212	7052	11767.02917
13	M	11	0.021623	1.81911	5.873083	0.553902	23604617533	2791	8269	11171.26604
14	M	11	0.021818	1.808244	4.107169	0.557361	20026042760	3296	8151	11529.18369
15	M	11	0.0233	1.73074	4.679972	0.586656	23229623645	2173	7812	11604.16095
16	M	11	0.021129	1.847364	5.873396	0.501963	14981982453	2076	7715	11639.14843
17	F	11	0.021638	1.818229	5.230039	0.547168	22758007010	2559	8402	11255.34982
18	F	11	0.02157	1.822056	5.320899	0.526924	24674810041	2434	8059	11412.49146
19	M	11	0.025801	1.616973	8.532931	0.649416	45613429480	3012	8861	12403.291
20	M	11	0.024132	1.690701	7.562027	0.629856	39580453194	2923	8058	12371.30956
21	F	11	0.018343	2.029972	4.426316	0.569182	32740511091	2061	6866	11769.49451
22	F	11	0.02061	1.878199	4.410363	0.555754	31864136284	2096	7132	11722.45473
23	M	12	0.020629	1.877081	7.389063	0.53558	47209854967	3113	7864	11964.71244
24	M	12	0.019845	1.926212	5.590317	0.543369	53028607188	3545	8097	12270.74622
25	F	12	0.020348	1.894333	6.518801	0.611594	37712680314	2755	8236	12220.38719
26	F	12	0.020974	1.856417	6.110044	0.614911	38344256047	2818	8029	12326.74408
27	M	12	0.01966	1.938233	4.304075	0.594326	29853032380	3905	8160	11622.38912
28	M	12	0.020374	1.892673	4.306742	0.628231	31033256513	4238	8160	11835.24814
29	F	12	0.020413	1.890303	6.372309	0.494665	22833296515	3808	7983	12338.6693
30	F	12	0.023671	1.712577	7.023733	0.509597	22900656575	3705	7400	12120.84312
31	M	12	0.022525	1.770206	6.607676	0.498085	36583842745	3352	7313	12316.14709
32	M	12	0.021971	1.79982	7.353841	0.559008	38744911016	3003	8420	12519.97747
33	M	13	0.021339	1.835181	6.168941	0.468707	29813277400	3637	7791	12352.46627
34	M	13	0.021459	1.828333	6.249716	0.488648	34136387870	3907	8676	12488.36907
35	F	13	0.018523	2.016798	5.275171	0.570821	39900346767	2850	7220	12022.89912
36	F	13	0.020577	1.880226	5.123482	0.624166	39160832422	2798	7843	11976.46695
37	M	13	0.021511	1.825405	4.057986	0.547199	21840672300	2903	7507	11941.28578
38	M	13	0.024447	1.676144	6.005678	0.601346	26611204397	3171	7982	12068.4718
39	F	13	0.02262	1.765256	9.397294	0.608222	37355526594	1790	8027	10909.20675
40	F	13	0.020992	1.855392	8.837594	0.591463	39589479130	2141	8470	11071.58652
41	F	14	0.022205	1.787161	5.262792	0.543985	22003092875	2483	6557	11258.15925
42	F	14	0.022486	1.772238	6.633677	0.549734	26463262291	2433	7576	11469.84675
43	F	14	0.021703	1.814612	4.827635	0.62714	29603682714	2733	8733	11561.1225
44	F	14	0.02234	1.779949	5.059524	0.568952	28520320675	2861	9116	11714.62255
45	F	14	0.020094	1.910212	4.138663	0.569064	23866877083	1943	7777	11423.90741
46	F	14	0.022482	1.772435	4.060924	0.560892	21095126948	2390	7837	11677.01562
47	M	14	0.022104	1.792588	6.376737	0.730289	36370861554	2635	7580	11998.73374
48	M	14	0.020482	1.88607	4.410588	0.639008	39681806515	2422	7869	11793.505
49	F	14	0.019911	1.921915	6.741353	0.565301	38795696083	2134	7463	11710.87846
50	F	14	0.019368	1.957697	6.175702	0.525199	42879707399	1871	7948	11842.78614
51	F	14	0.021894	1.804072	7.108724	0.553969	41430460602	1758	8430	11313.02218
52	F	14	0.022352	1.779343	8.490407	0.601104	39604644274	2723	9110	11484.29886
53	M	15	0.021029	1.85317	3.0375	0.552268	21472798067	2245	7154	11660.74345
54	M	15	0.019857	1.925441	3.0375	0.517124	13024360715	2736	7036	11937.37792
55	F	15	0.022966	1.74748	6.608635	0.555018	26858455233	3028	7836	11287.487
56	F	15	0.025004	1.651153	6.113471	0.573236	28540974765	3439	7597	11526.87026
57	F	15	0.022154	1.789921	5.200833	0.502361	22338808639	2257	7176	11441.67389
58	F	15	0.023333	1.729106	5.670729	0.459634	21877803048	1518	6512	10983.55823
59	M	15	0.023066	1.742395	8.103352	0.577772	34815995638	2936	7978	11478.29548
60	M	15	0.020068	1.911909	8.413208	0.556302	29364989280	2867	8070	11281.90101
61	F	15	0.019952	1.919277	3.119074	0.531197	16133302070	2927	7006	11397.11437
62	F	15	0.021984	1.799108	2.025	0.503369	10201767741	2790	6479	11031.25704
63	M	15	0.02302	1.744754	2.181252	0.541816	10753476882	2779	7438	11960.13856
64	M	15	0.025497	1.629824	2.191086	0.538068	8677532596	3430	8236	12428.60096
65	M	16	0.018103	2.047834	6.074074	0.43903	43542295803	2415	6893	11821.07361
66	M	16	0.01654	2.174947	6.167338	0.432799	37528583640	1977	7761	11558.69057
67	F	16	0.024288	1.683475	8.101449	0.61634	20178129093	2488	7476	11460.99992
68	F	16	0.021584	1.82127	6.018705	0.609051	19518627704	2057	6800	10739.49665
69	M	16	0.019573	1.943989	3.079389	0.526097	20832490861	2941	7220	12006.95146
70	M	16	0.01802	2.054149	2.143159	0.450451	17266841825	3164	7253	11906.38755
71	M	16	0.020827	1.865145	4.522712	0.51395	20839344896	3396	8438	11945.72789
72	M	16	0.020272	1.899059	5.578153	0.452693	22652779778	3173	8002	11706.8126
73	M	16	0.025281	1.639089	4.200131	0.593535	18800681481	4077	7631	12091.45429
74	M	16	0.021486	1.82681	3.068502	0.546037	20243453763	3437	8067	12231.51215
75	F	16	0.022174	1.788816	4.090627	0.610696	27461048610	2742	7183	11187.13482
76	F	16	0.021656	1.817257	5.164016	0.545525	31866280377	2677	7802	11176.6375
77	M	16	0.023597	1.716187	4.687152	0.681302	22252201452	3726	7888	11646.01712
78	M	16	0.024976	1.652404	6.039282	0.68612	23161742701	3028	7771	11471.97034
79	M	17	0.02262	1.76523	2.026667	0.482851	9991750303	2399	8147	12637.50367
80	M	17	0.02346	1.722834	4.226484	0.561137	18416432917	3274	8164	12639.55509
81	M	17	0.020825	1.865253	6.890427	0.445	31141518464	1945	7109	11705.0495
82	M	17	0.019706	1.935226	6.305925	0.489256	40102535123	1841	8307	11938.38681
83	F	17	0.022461	1.773553	4.275915	0.556712	23081762339	3424	7668	11969.41465
84	F	17	0.024396	1.678517	5.114538	0.590641	25304687547	3164	8840	12215.72157
85	M	17	0.019484	1.949901	6.132507	0.507619	37949751776	3137	7724	11433.48672
86	M	17	0.01895	1.98635	7.193891	0.427588	30127917386	2999	8018	11683.9834
87	F	17	0.019877	1.924151	5.579893	0.501001	40248406225	2970	8070	12103.23063
88	F	17	0.020382	1.892223	6.507505	0.508887	44854671152	2093	7581	11908.32885
89	F	18	0.020541	1.882429	4.107499	0.554954	19500225791	3824	9057	11947.39621
90	F	18	0.021174	1.844708	3.073018	0.539139	13982731376	3800	8888	11977.70219
91	F	18	0.020051	1.912988	5.251952	0.462725	24860092293	2376	8229	11849.596
92	F	18	0.017291	2.111445	5.464645	0.476029	25296366108	1862	9240	11876.91579
93	M	18	0.020056	1.912682	6.10529	0.463661	42203709072	2412	7053	11663.04647
94	M	18	0.019792	1.929649	7.205491	0.477014	41463023401	2164	7739	11657.92101
95	M	18	0.022106	1.792519	6.115557	0.531074	35237116273	3552	8255	11979.306
96	M	18	0.022929	1.749336	6.743762	0.524023	37201461851	3669	8197	11884.56229
97	F	18	0.02225	1.784754	5.735984	0.618546	27162405793	2507	7402	12023.4374
98	F	18	0.018629	2.009131	5.580966	0.474123	28120556247	1803	7560	11991.27314
99	M	18	0.020572	1.880555	7.145392	0.462924	30883468350	2383	7660	10818.31824
100	M	18	0.019952	1.91928	6.568114	0.470506	33257819995	3082	7577	11188.38532
101	F	18	0.022562	1.76828	6.31974	0.597092	32105677373	1993	6806	10730.34463

Patient	Percentile_25	Percentile_50	Percentile_75	Grad_Mean	Grad_Std	GLCM_ASM	GLCM_IDM	GLCM_IDMN	GLCM_Homogeneity	GLCM_Contrast	GLCM_Entropy	GLCM_DiffAverage
1	5435	5847	6259	475.200305	201.204907	0.062218	0.554963	0.9995	0.592324	2.051866	4.434698	1.078132
2	5238	5668	6071	521.884592	229.367057	0.056121	0.545554	0.999489	0.584684	2.095915	4.58653	1.102492
3	4858	5356	5810	535.567441	239.417662	0.037597	0.465189	0.999115	0.522167	3.635227	5.147175	1.464132
4	5303	5749	6212	506.776785	232.540258	0.042621	0.491198	0.999272	0.541744	2.98886	4.990466	1.328279
5	4224	4666	5142	640.807518	286.352085	0.035827	0.461859	0.999111	0.519302	3.651353	5.224923	1.475728
6	4441	4925	5418	663.09581	332.889368	0.03708	0.472532	0.999209	0.527141	3.249831	5.158231	1.399579
7	4210	4690	5126	543.890584	247.125088	0.050964	0.538987	0.999459	0.579937	2.221077	4.759372	1.131954
8	3947	4473	5017	587.822188	266.237737	0.042237	0.536531	0.999427	0.578333	2.352453	4.974023	1.155295
9	4963	5512	6078	605.739075	269.900334	0.031968	0.457084	0.999127	0.515444	3.585598	5.365481	1.475429
10	4679	5255	5813	626.42364	305.326054	0.030486	0.450616	0.999047	0.510203	3.916669	5.46664	1.530129
11	4933	5344	5755	455.633826	204.004065	0.0582	0.556798	0.999515	0.593383	1.989856	4.551895	1.065005
12	4568	4997	5417	486.024248	225.442446	0.05684	0.569276	0.99954	0.60396	1.888312	4.584197	1.028691
13	4701	5256	5795	715.205091	317.666399	0.027817	0.448484	0.999037	0.508528	3.957053	5.616392	1.537471
14	5105	5660	6165	685.14248	305.379699	0.02897	0.435339	0.998941	0.498751	4.353486	5.553795	1.615403
15	4346	4915	5493	682.648577	301.799632	0.026034	0.421567	0.998819	0.487295	4.854373	5.721585	1.704316
16	4370	4964	5550	722.274612	361.844892	0.024669	0.421248	0.998782	0.487843	5.009149	5.782977	1.720068
17	4599	5284	5903	677.073229	308.846912	0.024824	0.470083	0.999158	0.525775	3.458214	5.732011	1.431113
18	4916	5443	5953	657.830444	285.737446	0.031423	0.452348	0.999042	0.512115	3.934856	5.429441	1.52742
19	4959	5502	6095	657.981148	303.777635	0.030634	0.480295	0.99914	0.534075	3.53474	5.497351	1.417748
20	4991	5535	6054	629.73444	317.081515	0.036837	0.517577	0.999351	0.563148	2.663041	5.188414	1.234209
21	4043	4548	5079	652.565158	295.120034	0.042735	0.540307	0.999461	0.580512	2.211239	4.966056	1.128329
22	3928	4424	4999	614.29602	290.482052	0.038284	0.518002	0.999372	0.563668	2.577743	5.154073	1.221782
23	5131	5579	6019	491.70192	218.586549	0.04507	0.527564	0.999412	0.570637	2.414225	4.912473	1.18029
24	5433	5881	6330	506.331543	227.616195	0.042354	0.521642	0.999393	0.566235	2.489775	5.00586	1.202304
25	4874	5351	5910	564.609771	242.283776	0.040704	0.538412	0.999449	0.579312	2.259392	5.031105	1.138103
26	4919	5468	6018	520.599301	230.782958	0.038372	0.529443	0.999436	0.572795	2.31328	5.095414	1.163303
27	5472	5935	6447	647.379902	322.818376	0.037973	0.508412	0.999326	0.5559	2.765266	5.1361	1.265512
28	5724	6179	6634	586.195079	257.765743	0.037603	0.51117	0.999359	0.55853	2.632683	5.129153	1.242767
29	5299	5780	6294	546.651874	259.375356	0.045052	0.545641	0.999462	0.585458	2.207396	4.858924	1.118316
30	5137	5574	6020	476.10317	204.041046	0.049925	0.55563	0.999517	0.593176	1.980133	4.70355	1.066819
31	5013	5456	5941	489.731349	216.445096	0.047388	0.546586	0.99948	0.586038	2.135325	4.803361	1.105839
32	5166	5669	6163	555.884643	245.407446	0.042143	0.53283	0.999434	0.574733	2.322098	5.012135	1.157714
33	5612	5956	6308	413.766018	201.194779	0.064584	0.565471	0.999543	0.60094	1.872587	4.391679	1.032987
34	5669	6095	6505	472.609381	222.366822	0.04896	0.540773	0.999453	0.581174	2.24449	4.875265	1.131891
35	4596	5044	5518	566.60942	264.559619	0.049046	0.550727	0.999497	0.589011	2.063737	4.770028	1.087404
36	4412	4921	5509	715.61542	319.717031	0.036733	0.508728	0.999334	0.556462	2.733193	5.234554	1.260607
37	4795	5270	5753	626.951839	297.515007	0.045577	0.543881	0.999472	0.583569	2.164984	4.86991	1.11506
38	4890	5347	5866	629.126994	283.115832	0.039932	0.514551	0.999361	0.561047	2.622105	5.063513	1.235188
39	4039	4783	5602	764.496883	337.907208	0.018288	0.423075	0.998866	0.488951	4.661242	6.145894	1.67974
40	4315	4975	5703	742.271059	347.219173	0.019753	0.415284	0.998781	0.482399	5.013567	6.06264	1.737499
41	3936	4353	4796	576.620786	294.528949	0.04971	0.513516	0.999371	0.559424	2.581832	4.722288	1.229092
42	4078	4553	5063	665.79055	315.254469	0.040883	0.494733	0.999269	0.54561	2.999605	5.086174	1.322318
43	5422	5972	6563	831.870452	398.067136	0.027036	0.468679	0.999101	0.52456	3.693657	5.668887	1.464079
44	5637	6148	6635	717.880169	369.813719	0.032027	0.476198	0.999154	0.530268	3.475171	5.449812	1.419316
45	4163	4731	5290	756.002954	323.415183	0.028678	0.445509	0.999012	0.506944	4.058693	5.588633	1.558238
46	4447	4998	5540	688.309028	319.530331	0.030332	0.430564	0.998905	0.494976	4.502069	5.481848	1.645359
47	4652	5171	5716	523.386714	250.450699	0.036846	0.527793	0.99943	0.571187	2.339208	5.200196	1.170431
48	4414	4959	5495	555.963979	269.802798	0.038434	0.535496	0.999452	0.576914	2.247747	5.109713	1.142655
49	4047	4633	5262	658.606838	284.185433	0.031645	0.498653	0.999292	0.548409	2.906305	5.389216	1.303449
50	4208	4794	5406	742.313436	315.43566	0.02991	0.483673	0.999203	0.536673	3.270914	5.471561	1.381811
51	4393	4961	5546	681.512133	304.030186	0.030977	0.501799	0.999311	0.5506	2.829317	5.457164	1.286286
52	4479	5073	5753	664.776989	314.592239	0.028378	0.498382	0.999279	0.548132	2.961384	5.571429	1.310375
53	4480	4852	5208	440.434623	193.311774	0.060379	0.546127	0.999485	0.584842	2.113983	4.526476	1.102747
54	4623	5140	5605	524.628251	210.093884	0.042813	0.507911	0.999345	0.555551	2.687364	4.977042	1.256343
55	4798	5333	5868	655.659935	299.748313	0.031301	0.484203	0.999211	0.536342	3.241324	5.420779	1.374178
56	5102	5555	6033	572.722364	262.841262	0.039208	0.49368	0.999272	0.544306	2.990113	5.078945	1.32498
57	4183	4654	5141	715.472473	341.402306	0.041601	0.517048	0.999339	0.563042	2.712225	5.070067	1.24232
58	3729	4225	4696	747.020851	377.730142	0.038746	0.487551	0.999251	0.539616	3.074416	5.174045	1.346951
59	4950	5411	5903	553.046143	229.388953	0.035059	0.504582	0.999326	0.552922	2.764927	5.254642	1.273006
60	4757	5203	5680	609.510452	297.590473	0.039571	0.511715	0.999352	0.558283	2.660871	5.133252	1.243775
61	4403	4914	5417	733.304674	315.041074	0.038047	0.509797	0.999326	0.557165	2.765961	5.069438	1.262347
62	4103	4500	5003	595.301073	264.4517	0.045089	0.513834	0.99938	0.560058	2.543679	4.818014	1.225221
63	4531	5095	5599	719.587852	330.513733	0.03992	0.521194	0.999386	0.566164	2.51832	5.041778	1.206884
64	4957	5573	6119	749.828831	351.293195	0.031054	0.447167	0.998947	0.50706	4.329907	5.408164	1.588903
65	4220	4720	5237	517.254516	218.920582	0.042374	0.538078	0.999461	0.578776	2.212268	4.934808	1.132727
66	3931	4413	4904	563.951497	274.83413	0.046432	0.546318	0.999489	0.585261	2.096564	4.921252	1.100668
67	4238	4762	5371	726.039975	362.735533	0.032639	0.486002	0.999224	0.538973	3.18784	5.352494	1.367442
68	3638	4086	4551	865.013188	526.918311	0.043865	0.490775	0.999242	0.542215	3.113321	4.951324	1.345942
69	4667	5047	5419	544.249377	245.83534	0.058283	0.536576	0.999424	0.577791	2.364508	4.583172	1.155871
70	4560	4940	5344	577.260831	262.004803	0.056512	0.532668	0.999418	0.575101	2.387277	4.58384	1.165769
71	5262	5784	6272	619.953026	339.370565	0.040412	0.541963	0.999447	0.582962	2.270352	5.097797	1.134549
72	5014	5519	6007	601.777273	261.170239	0.036714	0.512934	0.999353	0.559157	2.656371	5.199257	1.241691
73	5470	5803	6187	432.132144	187.363046	0.061072	0.532884	0.999419	0.575886	2.382969	4.486942	1.166684
74	5444	5948	6469	527.496142	243.6065	0.038945	0.514123	0.999349	0.560339	2.672877	5.13015	1.2418
75	4527	4934	5358	574.279531	285.87435	0.045552	0.512682	0.999359	0.559397	2.630145	4.892956	1.239413
76	4502	4909	5341	612.269422	311.890385	0.044187	0.511255	0.999333	0.558108	2.738344	5.014228	1.254478
77	5213	5769	6318	665.589879	305.89874	0.032751	0.502843	0.999312	0.551748	2.822343	5.284125	1.284656
78	5146	5612	6060	657.274345	346.698302	0.04126	0.518593	0.999373	0.563685	2.574027	5.038421	1.219535
79	5368	6168	6655	582.430161	260.037527	0.036314	0.491527	0.999291	0.542184	2.91241	5.300382	1.318115
80	5352	5987	6556	563.14321	231.244628	0.031255	0.496524	0.999271	0.546705	2.995206	5.430203	1.317003
81	4048	4652	5221	669.223234	327.181043	0.031705	0.510105	0.999316	0.557355	2.809377	5.405453	1.266525
82	4143	4867	5540	778.796267	339.566435	0.023965	0.456821	0.999071	0.515751	3.815567	5.8127	1.505703
83	4998	5500	5994	663.331437	321.801072	0.037475	0.485119	0.999217	0.537749	3.215228	5.149985	1.370614
84	5207	5693	6245	731.7658	360.264188	0.035013	0.493544	0.999215	0.544289	3.22376	5.338958	1.353547
85	4848	5331	5806	491.975812	209.083534	0.041405	0.530911	0.99943	0.573848	2.33974	5.020673	1.164939
86	5150	5616	6100	514.273943	250.316385	0.039987	0.514379	0.999365	0.561143	2.605703	5.13495	1.232342
87	4715	5119	5541	500.88274	225.183137	0.05452	0.551547	0.999488	0.589701	2.102742	4.663279	1.091714
88	4453	4959	5440	550.154882	255.377654	0.039982	0.518639	0.999376	0.56394	2.560373	5.1029	1.218538
89	5701	6194	6655	642.84165	339.450689	0.037041	0.489918	0.999241	0.541295	3.116405	5.236271	1.348052
90	5741	6243	6744	607.438345	304.747647	0.03463	0.483448	0.99917	0.535623	3.407951	5.387311	1.39663
91	4200	4772	5360	877.475825	435.609208	0.031296	0.467693	0.999048	0.52437	3.912515	5.480427	1.490623
92	4109	4697	5385	911.014554	467.550506	0.027754	0.46066	0.999075	0.518306	3.800805	5.730749	1.49329
93	4077	4574	5089	649.869006	322.102793	0.042602	0.528942	0.999412	0.57199	2.412477	5.020655	1.178146
94	4085	4583	5072	653.833585	287.115712	0.041895	0.510875	0.999352	0.557668	2.65781	5.061806	1.245882
95	5463	5912	6286	416.650201	175.102621	0.048441	0.542043	0.999471	0.581935	2.170399	4.833498	1.119222
96	5231	5721	6261	561.459037	261.950811	0.036583	0.527357	0.999427	0.570271	2.350608	5.156261	1.171886
97	4493	4994	5486	802.304105	477.755291	0.043156	0.515215	0.999364	0.560803	2.611666	4.98799	1.230651
98	4414	4976	5530	787.418576	419.106298	0.032431	0.463604	0.999095	0.52053	3.716557	5.393783	1.477588
99	4274	4859	5489	561.960777	277.42705	0.026799	0.497693	0.999309	0.547442	2.837041	5.600177	1.296414
100	4775	5223	5747	518.79059	295.532826	0.034148	0.504243	0.999317	0.552587	2.804576	5.303397	1.278744
101	3936	4351	4765	549.247669	274.762562	0.046055	0.512751	0.999362	0.559376	2.617821	4.897198	1.237725

Patient	GLCM_DiffVariance	GLRLM_SRHGE	GLRLM_LRLGE	GLRLM_RP	GLRLM_RV	GLRLM_RE	GLSZM_LAHGLE	NGTDM_Strength	GLDM_SDHGLE
1	0.868906	7.104503	0.000038	0.012575	0.02518	0.114275	304.356948	0.137438	72.6167
2	0.846495	4.491114	0.000028	0.008251	0.018268	0.080689	316.710875	0.137595	72.629567
3	1.442115	9.015138	0.000045	0.016193	0.027318	0.142041	197.830396	0.186637	78.374151
4	1.199983	13.011132	0.000062	0.022607	0.039999	0.189719	266.247392	0.155174	69.402889
5	1.436204	5.639381	0.00003	0.010827	0.017486	0.101737	182.458904	0.250893	81.983903
6	1.256573	4.363198	0.000022	0.007893	0.013267	0.077494	173.815013	0.23475	82.355336
7	0.913534	10.443298	0.000092	0.023111	0.049213	0.198777	347.291759	0.093605	41.487624
8	0.98432	11.435342	0.000109	0.025883	0.056388	0.223766	404.650844	0.087093	38.926808
9	1.371989	6.933435	0.000036	0.012636	0.022215	0.120454	206.248936	0.13566	70.405038
10	1.533563	9.563358	0.000055	0.01823	0.032165	0.164396	200.543733	0.119841	74.302012
11	0.829177	8.906336	0.00007	0.018406	0.040753	0.162562	382.902582	0.075651	41.102864
12	0.808644	8.947979	0.000082	0.019744	0.045216	0.173903	465.549561	0.115554	36.8845
13	1.545048	7.174692	0.000038	0.013207	0.023264	0.126975	188.812655	0.2174	76.177346
14	1.690654	8.642518	0.000036	0.014584	0.023143	0.134646	177.897126	0.15603	89.155801
15	1.897456	6.508934	0.000031	0.011724	0.019041	0.113231	141.098972	0.20602	87.28565
16	2.006718	3.140751	0.000015	0.005568	0.009146	0.059631	126.86553	0.324487	111.540705
17	1.366095	7.087986	0.000037	0.012921	0.022166	0.127027	220.522368	0.293173	74.061287
18	1.545077	7.782661	0.000037	0.013599	0.023622	0.127734	217.19871	0.204169	72.199696
19	1.462372	14.098788	0.00007	0.024025	0.047312	0.213635	271.474503	0.17412	64.402792
20	1.083771	18.592001	0.000101	0.032136	0.066985	0.270546	355.734258	0.142809	57.377457
21	0.902192	11.770626	0.000102	0.02576	0.053587	0.221215	344.104338	0.104312	41.544697
22	1.040269	8.937901	0.000078	0.01975	0.04024	0.177267	325.928074	0.131679	42.875158
23	0.988816	9.658229	0.000061	0.018091	0.038814	0.163866	307.288206	0.091442	52.360707
24	1.013942	11.384223	0.000065	0.020152	0.043088	0.179484	316.598291	0.083461	55.137224
25	0.930642	8.273556	0.00006	0.01645	0.035689	0.153152	381.668113	0.161998	46.06393
26	0.930287	7.99289	0.000057	0.015756	0.034141	0.148674	349.724435	0.120394	49.732064
27	1.103713	13.31292	0.000064	0.021974	0.044401	0.193154	335.92615	0.132213	64.348804
28	1.039798	13.034783	0.000057	0.020417	0.041857	0.182422	394.445913	0.103154	64.692107
29	0.910815	6.755405	0.000039	0.012077	0.026446	0.116681	445.166045	0.160715	56.646904
30	0.813697	9.302433	0.000062	0.01741	0.040225	0.158502	397.113879	0.116768	51.176235
31	0.889665	7.972137	0.00005	0.014725	0.03264	0.137544	413.974209	0.098464	48.986623
32	0.952956	8.731501	0.000051	0.015537	0.034046	0.145567	323.342795	0.155294	56.780025
33	0.787451	10.53221	0.000057	0.017856	0.041151	0.156711	413.652857	0.125121	56.733827
34	0.937393	10.789014	0.000049	0.017458	0.036219	0.15781	390.770781	0.137991	61.073567
35	0.860573	9.025814	0.000065	0.018073	0.038153	0.162531	372.593	0.089085	46.968602
36	1.096956	9.950716	0.00007	0.019815	0.039972	0.178929	326.568647	0.112247	51.742635
37	0.894701	9.07201	0.000058	0.01725	0.035205	0.156113	369.061837	0.205361	53.472301
38	1.047118	7.812861	0.000051	0.014739	0.032001	0.137676	371.327381	0.161699	52.956885
39	1.771917	5.597522	0.000032	0.010876	0.018148	0.111803	173.198378	0.154036	71.525978
40	1.916873	5.577705	0.00003	0.010458	0.017449	0.106597	170.039556	0.165015	80.35718
41	1.029658	6.066534	0.000045	0.012886	0.025003	0.11902	259.380597	0.09003	52.8342
42	1.202182	7.289482	0.000053	0.014951	0.029956	0.138052	290.455471	0.135562	53.573656
43	1.470012	11.348615	0.000044	0.017604	0.032388	0.164292	234.911336	0.292717	75.3403
44	1.403743	9.944005	0.000036	0.015032	0.027084	0.140756	232.026222	0.355805	77.392603
45	1.588093	8.465933	0.00005	0.01677	0.02789	0.15353	159.921665	0.206957	75.218992
46	1.739157	8.270141	0.000044	0.015518	0.025282	0.140707	169.471014	0.178765	79.153694
47	0.935255	11.329829	0.000079	0.021953	0.047588	0.197969	348.924259	0.122829	48.52825
48	0.917514	13.186007	0.000091	0.026151	0.052967	0.227101	354.052632	0.145705	47.067321
49	1.162136	7.701366	0.000053	0.01551	0.030214	0.147789	294.829609	0.131064	49.214618
50	1.314764	7.670703	0.000049	0.015081	0.028022	0.143052	247.265677	0.141621	60.640436
51	1.138552	6.673621	0.000048	0.013584	0.027784	0.133108	294.42144	0.216567	51.063857
52	1.207384	7.825411	0.000052	0.015455	0.030558	0.149721	301.649293	0.214435	53.851439
53	0.881459	7.808212	0.000052	0.015502	0.030423	0.136906	338.186242	0.192598	55.641165
54	1.072858	3.638485	0.00002	0.006668	0.012672	0.067832	240.318653	0.251205	73.674733
55	1.299171	7.808765	0.000044	0.014205	0.027767	0.136209	272.42029	0.162694	63.599199
56	1.186718	9.107664	0.000047	0.015982	0.031181	0.146232	289.513253	0.123839	60.648879
57	1.120408	7.14062	0.000054	0.014816	0.03075	0.138116	336.630769	0.21158	48.126453
58	1.213852	5.409121	0.000042	0.011728	0.022457	0.111915	254.728395	0.14615	51.460494
59	1.100771	10.968575	0.000051	0.018198	0.034939	0.167066	303.056936	0.169197	58.295909
60	1.073474	8.30175	0.000044	0.01441	0.029584	0.136119	310.217391	0.199473	65.490153
61	1.112602	9.821698	0.000057	0.018238	0.034329	0.161817	302.390295	0.186431	62.174954
62	0.986614	5.12543	0.000037	0.01033	0.021285	0.098173	250.209913	0.205632	65.399328
63	1.019969	6.624574	0.000039	0.012624	0.02284	0.117318	247.595469	0.404403	66.438108
64	1.661797	4.071454	0.000023	0.007174	0.014504	0.072635	187.118577	0.451686	92.701188
65	0.902498	5.457567	0.000043	0.011504	0.024144	0.112289	359.16095	0.076432	45.746001
66	0.864732	4.990816	0.000043	0.011081	0.022997	0.108356	319.676329	0.150927	47.736204
67	1.258362	10.067373	0.000063	0.019919	0.036148	0.178919	273.240132	0.181015	59.676706
68	1.243307	7.626313	0.000068	0.017284	0.034086	0.152901	247.591654	0.162681	52.102629
69	0.997951	11.459383	0.000073	0.021495	0.044649	0.178573	261.963028	0.153888	60.489144
70	0.992951	8.24097	0.000053	0.015438	0.032432	0.134432	314.165254	0.182087	67.952393
71	0.954298	7.341692	0.000043	0.013305	0.028675	0.128521	305.258993	0.272781	60.107763
72	1.074277	6.25987	0.000035	0.011444	0.02215	0.112032	299.485179	0.179478	60.575471
73	0.986488	11.056166	0.000058	0.019439	0.038709	0.164152	338.002049	0.092221	58.52683
74	1.090473	10.71253	0.000051	0.017914	0.034355	0.161696	345.005792	0.220061	68.865162
75	1.058181	12.320616	0.000089	0.02412	0.052655	0.205173	313.393976	0.123821	55.9148
76	1.133459	9.722704	0.000063	0.018882	0.037374	0.167487	343.266596	0.13846	57.668309
77	1.115998	14.293754	0.000076	0.024551	0.050346	0.215228	335.521669	0.142476	60.551151
78	1.053898	11.509179	0.000067	0.020827	0.043098	0.184718	323.45	0.179486	55.61014
79	1.139439	4.72466	0.000019	0.007162	0.013674	0.07357	135.749104	0.856905	93.662154
80	1.224777	6.147617	0.000025	0.009612	0.018148	0.096882	231.710583	0.257107	76.945749
81	1.165455	6.312937	0.000048	0.01323	0.026765	0.129644	325.258621	0.13047	51.956581
82	1.493108	8.112276	0.000052	0.015858	0.029921	0.153143	221.496491	0.179046	59.697298
83	1.277768	7.193153	0.000036	0.012625	0.023758	0.1199	247.740506	0.128443	60.491341
84	1.334979	8.132135	0.000044	0.014074	0.029538	0.133897	293.051593	0.259078	70.973313
85	0.959658	6.78101	0.000046	0.013317	0.027813	0.127039	339.841182	0.101266	54.312132
86	1.057695	5.521192	0.000032	0.010177	0.020285	0.100948	295.4777	0.165074	61.849314
87	0.88666	8.865724	0.000067	0.017446	0.040973	0.156711	396.344546	0.11248	46.722796
88	1.038791	8.602755	0.00006	0.016926	0.036079	0.156794	333.84329	0.115654	47.227613
89	1.262452	7.340011	0.000027	0.011144	0.020596	0.107665	226.007181	0.304623	88.905009
90	1.41388	7.37169	0.000026	0.011007	0.019431	0.10639	204.448357	0.434795	94.888347
91	1.615708	5.127547	0.00003	0.00975	0.018313	0.097429	245.407913	0.239366	70.48893
92	1.507859	3.935278	0.000023	0.007494	0.013428	0.078716	200.239544	0.450346	86.188347
93	0.990011	7.077891	0.000058	0.015068	0.032344	0.141726	359.243816	0.076063	44.847883
94	1.06939	6.96726	0.000055	0.014716	0.030278	0.13728	317.342382	0.11784	45.613861
95	0.899065	8.496868	0.000044	0.014545	0.030567	0.135149	343.736783	0.124067	55.342449
96	0.946598	8.726435	0.000048	0.015154	0.032623	0.144569	348.165975	0.121087	54.05374
97	1.059733	8.37449	0.00006	0.017011	0.034377	0.154497	253.006812	0.116022	56.699974
98	1.453148	4.635321	0.00003	0.009172	0.017523	0.09214	228.964634	0.194893	68.700609
99	1.118104	6.339527	0.000034	0.011324	0.021719	0.114696	311.518738	0.175938	57.477777
100	1.137685	7.63245	0.000039	0.013036	0.026882	0.126888	292.013436	0.105664	68.28816
101	1.056124	8.866526	0.000071	0.019007	0.038904	0.16758	306.29195	0.099291	47.454354

102	F	18	0.021275	1.838852	6.887759	0.617394	32150731127	1749	6920	10720.36255	3863	4335	4839	678.833117	316.304643	0.035916	0.485951	0.999227	0.538282	3.175585	5.237328	1.363815	1.267824	9.195109	0.000067	0.019257	0.036147	0.172472	274.848569	0.121405	50.991395
103	F	19	0.020073	1.911595	5.481638	0.465387	20625365687	2904	8108	11682.10367	4475	5056	5735	876.668738	469.881935	0.026153	0.472977	0.999095	0.527504	3.718105	5.699277	1.454124	1.530336	5.428265	0.000029	0.009845	0.018091	0.100129	253.241042	0.247281	72.786482
104	F	19	0.021167	1.845128	3.038461	0.465272	12831067491	2634	8026	11662.01807	4540	5072	5596	1065.075641	587.473001	0.035795	0.493355	0.999289	0.54304	2.919014	5.267142	1.312214	1.143631	4.774643	0.000026	0.008786	0.015883	0.087814	212.5775	0.480986	84.191727
105	F	19	0.019431	1.953426	3.164193	0.504469	12600575200	2708	6734	11450.26574	4313	4729	5187	586.48948	278.022397	0.044839	0.507302	0.999344	0.554014	2.692588	4.901802	1.256696	1.083386	4.413139	0.000024	0.008457	0.014028	0.081428	206.046798	0.225794	75.812373
106	F	19	0.020792	1.867273	4.670239	0.610493	23241480550	2308	7932	11447.1014	4255	4671	5145	597.885669	363.040073	0.045306	0.503211	0.999276	0.551811	2.971958	5.028248	1.301852	1.230935	9.646749	0.000066	0.019227	0.037353	0.16738	274.393895	0.244757	59.261388
107	F	19	0.0224	1.776756	3.112028	0.521045	11930223402	3011	7758	11838.53951	4942	5468	5979	660.839693	312.737939	0.032572	0.487758	0.999234	0.540208	3.14625	5.351321	1.356723	1.260014	6.293177	0.000025	0.010043	0.017067	0.098176	188.753388	0.399118	88.864043
108	F	19	0.02497	1.65266	3.086012	0.5739	9849249422	3769	8141	12208.99294	5293	5843	6362	552.895336	242.303107	0.029253	0.461474	0.999092	0.518568	3.730741	5.475372	1.482307	1.472966	5.213117	0.000017	0.007786	0.012141	0.078541	144.521595	0.30368	105.359852
109	M	19	0.01848	2.019914	4.169165	0.483155	31107037134	1841	7268	11329.33283	4259	4850	5408	815.141419	380.195739	0.031275	0.485599	0.999242	0.53739	3.113475	5.412575	1.35487	1.223741	5.931212	0.000037	0.011653	0.021206	0.114081	243.981366	0.180107	58.72048
110	M	19	0.019124	1.9743	3.084517	0.535356	20920083791	2974	7945	11880.07712	4834	5366	5916	776.250663	377.408851	0.032381	0.504933	0.99928	0.552998	2.956443	5.405377	1.296376	1.218593	5.007756	0.000025	0.008828	0.016163	0.089488	260.092905	0.252175	64.549337
111	F	19	0.020964	1.857026	4.692102	0.544637	19063471412	2208	6935	10824.33932	3975	4490	5022	693.285779	344.129616	0.032325	0.481581	0.999199	0.534784	3.291613	5.362538	1.38612	1.333144	9.002587	0.000058	0.018277	0.03173	0.165335	249.457778	0.162947	65.169188
112	F	19	0.020981	1.856025	4.193177	0.532869	21037770125	1914	7524	10754.97586	3844	4392	4997	935.349981	440.695695	0.025962	0.437973	0.998993	0.500242	4.137226	5.685572	1.58368	1.569616	6.605092	0.00004	0.01329	0.021763	0.126764	157.832905	0.230552	68.776253
113	M	20	0.026452	1.590364	3.672379	0.539085	8847409077	2591	8327	12365.68661	5144	5589	6001	729.551401	356.472087	0.049371	0.494795	0.999284	0.545497	2.937873	4.790422	1.314712	1.181856	6.317305	0.000032	0.010771	0.02108	0.100023	238.984791	0.627769	104.350583
114	M	20	0.021764	1.811225	3.239913	0.522062	16576051102	3020	7924	12455.04553	5212	5648	6102	545.956654	233.004181	0.045132	0.498252	0.999307	0.547943	2.845038	4.924834	1.295908	1.132436	8.662827	0.00004	0.014583	0.026817	0.130318	228.586283	0.248736	89.585234
115	M	20	0.018136	2.045333	4.033895	0.499174	22454401395	626	6436	10986.49738	2986	3564	4134	844.822998	450.090909	0.030779	0.455386	0.999079	0.514791	3.784089	5.433909	1.50544	1.471393	4.854571	0.000037	0.010871	0.018319	0.105292	223.48263	0.162546	67.434331
116	M	20	0.018099	2.048185	3.038754	0.439687	18149709074	1014	6287	10943.15484	2904	3506	4125	831.795903	391.064353	0.028249	0.461303	0.999126	0.519257	3.590561	5.509422	1.468105	1.386004	4.200337	0.000033	0.009463	0.016283	0.09449	194.293495	0.216994	68.990805
117	M	20	0.021183	1.844172	3.079148	0.531979	18135114323	2310	7815	12296.44247	4310	4846	5390	578.817896	263.279151	0.035117	0.478665	0.999193	0.53259	3.313673	5.319638	1.395758	1.334026	6.669681	0.000036	0.012474	0.021675	0.118376	209.41683	0.282024	74.139188
118	M	20	0.022762	1.757872	3.037975	0.523449	14884969437	2234	6879	12054.19176	4088	4590	5126	815.428962	423.837561	0.039725	0.498754	0.999302	0.548217	2.864882	5.069919	1.298748	1.152995	3.402188	0.000023	0.006807	0.013259	0.07001	227.393519	0.210356	74.804827
119	M	21	0.021343	1.834988	7.397193	0.477217	22209843152	1779	7481	11033.03098	4013	4539	5131	591.906986	281.697295	0.028486	0.470119	0.999186	0.526011	3.341382	5.592792	1.417865	1.295295	4.841104	0.000032	0.009505	0.01841	0.096477	204.545335	0.228021	66.574821
120	M	21	0.021642	1.818007	6.007139	0.470537	16188849332	2124	7448	11034.74148	4022	4605	5139	580.997102	277.632988	0.033641	0.505243	0.999298	0.553645	2.881286	5.329117	1.288594	1.191416	4.549768	0.000031	0.00911	0.017525	0.091811	247.077617	0.268374	66.987481
121	M	21	0.021278	1.83873	6.51908	0.60956	40757674848	3043	8645	11742.05094	4938	5517	6087	661.886203	273.735128	0.031411	0.509019	0.999348	0.556425	2.677477	5.408124	1.252547	1.084847	11.268141	0.000059	0.020004	0.0377	0.183125	329.77	0.201488	57.589733
122	M	21	0.01935	1.958909	5.466736	0.47529	20585965294	2959	7849	11876.84183	5215	5660	6112	620.717956	343.431716	0.042187	0.499544	0.999283	0.548524	2.943369	5.064237	1.30637	1.213007	5.443581	0.000027	0.009422	0.017838	0.092024	239.20742	0.237853	79.703139
123	F	21	0.022555	1.768619	9.113802	0.589761	49943146332	3287	7885	11146.94758	5000	5439	5925	585.086693	293.909422	0.037491	0.510203	0.999348	0.557293	2.676678	5.184889	1.249866	1.077349	10.093614	0.000054	0.017754	0.036154	0.16383	353.678351	0.090007	52.392407
124	F	21	0.02067	1.874573	5.135738	0.553472	21306771656	3350	7853	11046.9657	4849	5319	5893	1026.994446	690.320454	0.032824	0.496584	0.999282	0.547454	2.947561	5.36318	1.314364	1.165366	5.638648	0.00003	0.009926	0.019431	0.100161	282.472543	0.208422	65.996344
125	M	21	0.020092	1.910358	3.028846	0.559559	15351935155	3281	7500	11529.47491	4859	5282	5698	797.304156	434.901495	0.048832	0.519898	0.999388	0.565958	2.509476	4.808039	1.210756	0.993924	7.137671	0.000046	0.013181	0.028537	0.120719	231.374194	0.193493	66.758268
126	M	21	0.02231	1.78154	3.240366	0.530329	11072810607	2633	7998	11260.76725	4485	4983	5507	746.535819	353.22677	0.03644	0.503706	0.999302	0.552214	2.865423	5.25086	1.28831	1.140783	6.870614	0.000043	0.013272	0.025507	0.124948	217.198347	0.433322	67.867403
127	F	21	0.022749	1.758584	4.277234	0.561771	17457478464	1780	7408	12132.30698	4401	4869	5345	719.931188	435.101195	0.044364	0.508488	0.999332	0.55578	2.742197	4.971622	1.261394	1.113269	5.279027	0.000032	0.009909	0.020144	0.096741	241.729387	0.309043	67.278638
128	F	21	0.01963	1.940213	5.266378	0.463993	16120221870	2339	6709	11846.68803	4006	4499	5099	711.081265	407.224711	0.041314	0.521495	0.999403	0.565761	2.451411	5.022062	1.196741	0.99508	3.040895	0.000021	0.005875	0.012046	0.062253	250.856833	0.203023	64.348716
129	F	21	0.024528	1.672453	4.290009	0.520883	16617612750	3120	8235	11948.93279	4992	5549	6138	842.159637	432.322635	0.031598	0.487793	0.999239	0.539707	3.125828	5.367115	1.354654	1.221979	8.257341	0.000037	0.013504	0.025174	0.128241	311.359031	0.320856	69.694731
130	F	21	0.023717	1.710381	3.131434	0.544778	13677200960	3362	7614	11645.81431	4822	5250	5750	816.969732	410.20801	0.037911	0.472903	0.99916	0.527437	3.448014	5.14124	1.425028	1.35518	8.119042	0.000037	0.01405	0.024047	0.128054	218.78125	0.205353	80.737685
131	M	22	0.014649	2.358286	3.592302	0.45678	36367275432	2143	8191	11596.81604	4175	4643	5183	912.508885	561.587881	0.036593	0.507124	0.999307	0.554592	2.847032	5.260369	1.277433	1.182157	4.435621	0.000027	0.008459	0.016199	0.086527	280.835407	0.212127	66.155311
132	M	22	0.014371	2.388542	8.347845	0.415034	25577883487	1397	6889	11468.57058	4071	4587	5056	923.833157	500.71962	0.037598	0.494632	0.999277	0.545204	2.967755	5.196088	1.320023	1.183138	3.34553	0.000019	0.006368	0.011737	0.06668	213.434194	0.224864	70.024717
133	F	22	0.019991	1.916793	2.072324	0.471532	12671389457	3614	8887	12415.52981	5652	6376	7022	883.919708	391.825912	0.02226	0.459502	0.999031	0.518417	3.981429	5.850906	1.519708	1.57939	6.128802	0.000018	0.008467	0.014174	0.087426	208.273775	0.550552	104.134185
134	F	22	0.02147	1.8277	3.07721	0.554935	17132109428	4046	9021	12284.83155	5589	6171	6809	1007.930206	413.287568	0.026293	0.443413	0.999044	0.505544	3.925967	5.625266	1.549603	1.404261	9.271693	0.000029	0.013321	0.022472	0.12647	252.579618	0.38434	100.325124
135	F	22	0.022738	1.759132	6.911269	0.64445	32441999865	2907	7784	11506.97405	4797	5268	5747	517.523343	244.089687	0.036629	0.491827	0.999252	0.54281	3.071296	5.222736	1.337551	1.251129	9.920815	0.000053	0.01775	0.034087	0.160754	273.656155	0.107871	70.898479
136	F	22	0.020283	1.898343	7.737037	0.60911	27532717434	3548	8498	11869.01792	5179	5609	6136	721.51901	435.786606	0.037471	0.492707	0.999253	0.543502	3.069489	5.178984	1.333895	1.263425	7.62222	0.000036	0.012855	0.024425	0.121729	267.066919	0.175881	81.787056
137	M	22	0.021911	1.80313	6.01715	0.575053	31998391237	2883	8235	12090.55988	4833	5461	6097	825.771855	408.602899	0.027211	0.470153	0.999188	0.525407	3.334717	5.595382	1.416247	1.283022	9.108542	0.000042	0.015356	0.027655	0.145625	231.270179	0.164173	75.984688
138	M	22	0.022406	1.776458	5.115725	0.607074	23447761457	3634	8781	12486.83678	5371	5877	6383	610.745179	297.54816	0.03575	0.479976	0.999219	0.533403	3.204868	5.243138	1.380743	1.259157	8.080221	0.000035	0.013033	0.024676	0.123284	190.256289	0.213826	86.177601
139	F	22	0.02097	1.856641	7.761716	0.477353	41540471091	2652	7217	11461.48225	4122	4551	4988	619.119403	376.185765	0.047464	0.520293	0.999384	0.565123	2.528389	4.848684	1.209445	1.03643	6.292771	0.000045	0.012917	0.0261	0.121357	347.682692	0.09073	46.367069
140	F	22	0.017804	2.070731	7.758261	0.412919	33384427922	2322	7308	11776.68879	4345	4865	5409	656.571677	343.584975	0.037076	0.517473	0.999384	0.562691	2.529894	5.149097	1.215809	1.016617	5.565647	0.000035	0.010655	0.021032	0.105483	275.460526	0.124547	55.718251
141	F	22	0.025452	1.63175	4.06775	0.467895	7312729617	1829	7146	10828.88297	3837	4522	5137	1018.16841	459.683292	0.02705	0.467043	0.999059	0.523489	3.867844	5.577925	1.48753	1.533553	3.195603	0.000017	0.006056	0.010343	0.064409	253.958015	0.683665	84.516521
142	F	22	0.021922	1.802511	5.60035	0.531554	17270864666	2522	7402	11000.99091	4117	4569	5137	819.434236	427.453699	0.03565	0.507224	0.999294	0.554683	2.89984	5.278853	1.283337	1.201799	7.073611	0.000046	0.013629	0.027218	0.130269	327.372792	0.263377	69.981186
143	F	22	0.021846	1.806681	7.052037	0.568639	34600365053	2553	8737	11485.91833	4562	5060	5691	698.928876	403.758496	0.02997	0.475323	0.999173	0.529765	3.397392	5.558764	1.411006	1.340601	9.592776	0.000049	0.016721	0.031921	0.158018	241.956522	0.246012	67.957683
144	F	22	0.023098	1.740789	6.235796	0.66548	33163734527	2560	7615	11428.90099	4679	5146	5606	607.478858	302.287379	0.037773	0.486283	0.999228	0.538739	3.170613	5.187482	1.360952	1.275952	9.909966	0.000052	0.017244	0.035007	0.156478	281.574701	0.161767	69.128563
145	F	22	0.020438	1.888717	6.252899	0.560196	30134785866	1766	6425	10985.14374	3501	3981	4428	692.713011	420.112335	0.043813	0.509057	0.99935	0.556252	2.667091	4.923746	1.250217	1.070068	7.996118	0.000071	0.018287	0.035998	0.162768	304.733906	0.109703	48.07378
146	F	22	0.021877	1.804972	6.14636	0.60472	32841344306	1589	5742	10795.32539	3366	3804	4209	625.05691	389.969509	0.048867	0.51462	0.999355	0.560807	2.648794	4.805625	1.237618	1.088724	10.034488	0.0001	0.023916	0.048885	0.201481	302.566414	0.073355	44.861134
147	F	23	0.023837	1.704613	5.108478	0.682281	21552269546	3195	7117	11255.66172	4638	4972	5323	788.875446	592.274765	0.065944	0.547807	0.999448	0.587103	2.266826	4.412619	1.121623	0.980071	13.043572	0.000104	0.026116	0.062418	0.211177	352.254747	0.125808	58.055651
148	F	23	0.022782	1.756866	4.39932	0.669846	20104208824	2486	7174	11140.01967	4475	4891	5266	680.187652	385.688733	0.048201	0.513982	0.999363	0.560437	2.614292	4.861566	1.232925	1.05823	9.009909	0.000057	0.01749	0.034389	0.154461	257.357711	0.167878	58.447059
149	M	23	0.019918	1.92148	6.631881	0.515987	37286541377	2417	6770	11051.74848	4104	4514	4932	537.386265	281.230636	0.043984	0.485331	0.999234	0.537734	3.147217	4.990108	1.36169	1.266796	7.976386	0.000051	0.015689	0.030164	0.140918	219.133763	0.081415	64.823122
150	M	23	0.02049	1.885529	7.563206	0.496457	35805992834	1557	7014	11117.66106	4120	4577	5035	608.03338	355.539624	0.037054	0.471634	0.999175	0.526823	3.387658	5.219305	1.420276	1.330486	5.583362	0.000034	0.010753	0.02017	0.104339	224.613311	0.11511	69.112988
151	M	23	0.017796	2.071332	6.052397	0.447093	39839454944	2058	6894	11489.9634	4059	4575	5091	549.082516	262.501733	0.031896	0.461155	0.999152	0.518673	3.484272	5.389408	1.453832	1.340446	6.091628	0.000033	0.011374	0.0199	0.110545	207.464536	0.07988	71.160005
152	M	23	0.017946	2.059815	8.040717	0.4591	44378220786	1256	7061	11299.45603	3797	4369	4948	568.809251	263.364531	0.02861	0.469041	0.999169	0.52534	3.411985	5.532722	1.429489	1.347769	7.181076	0.000044	0.014049	0.025233	0.135218	227.006878	0.114962	68.562019
153	F	23	0.025295	1.638482	4.692102	0.582984	22050653358	2736	8213	12004.05504	4663	5119	5643	905.592746	468.994373	0.042378	0.512695	0.999308	0.559202	2.841706	5.080677	1.263155	1.185625	11.021603	0.000072	0.021148	0.043668	0.185401	398.534965	0.299412	62.159716
154	F	23	0.024563	1.670896	6.639928	0.557307	22191653828	2102	7038	11307.27697	3928	4469	5001	824.781986	381.750784	0.033425	0.483957	0.999213	0.53706	3.229904	5.337254	1.375769	1.270885	7.081874	0.000052	0.014886	0.028272	0.140195	283.43586	0.158414	54.272505
155	F	24	0.021036	1.852753	6.179315	0.530513	30746366135	2230	7540	11236.55348	4019	4643	5308	798.070426	381.378617	0.026973	0.49429	0.999283	0.544964	2.943209	5.608524	1.317159	1.168874	6.34728	0.000043	0.012572	0.024436	0.125762	267.008547	0.194249	57.258841
156	F	24	0.020901	1.860749	5.105398	0.494807	23318064901	2025	7401	11327.56339	4175	4815	5406	830.417889	381.771611	0.02915	0.495106	0.999282	0.546246	2.947874	5.509728	1.317841	1.151914	7.755899	0.000052	0.015121	0.030437	0.145428	324.477633	0.208692	60.099569
157	F	24	0.020848	1.863908	6.513206	0.466003	31790219754	2428	6988	11109.4019	4006	4427	4840	648.955045	406.338481	0.048333	0.522803	0.999383	0.567652	2.533169	4.849947	1.206525	1.037022	8.616419	0.00007	0.018357	0.038801	0.163167	328.018653	0.084766	47.535519
158	F	24	0.020394	1.891485	6.255643	0.50345	30053391421	2362	7781	11655.59726	4328	4939	5534	793.153239	407.804822	0.029733	0.490182	0.999251	0.541729	3.07389	5.490327	1.342587	1.223563	7.166926	0.000042	0.013471	0.025654	0.13108	272.01837	0.164657	60.665322
159	M	24	0.016491	2.179264	6.098361	0.422596	46330566162	2112	7286	10952.73283	3837	4266	4728	597.513398	349.995205	0.045427	0.52949	0.999423	0.572201	2.368197	4.905016	1.169051	0.977037	5.306845	0.000046	0.011648	0.025014	0.11253	346.279464	0.100286	45.97871
160	M	24	0.017708	2.078211	6.200315	0.451143	46227789292	1171	7278	10935.39868	3680	4282	4860	600.85488	301.857434	0.031803	0.523929	0.999404	0.567983	2.44411	5.389389	1.191754	0.99813	6.647514	0.000058	0.014552	0.030599	0.142276	338.025478	0.1527	46.29021
161	M	24	0.019069	1.97808	5.090212	0.499424	29829161513	2859	7210	11762.49668	4601	5030	5527	528.506788	292.093837	0.045054	0.552331	0.999505	0.590203	2.032378	4.846145	1.08054	0.845873	5.371856	0.000038	0.010592	0.02368	0.104863	312.669554	0.142912	50.504668
162	M	24	0.020721	1.871506	6.417471	0.566362	41688079157	2439	7142	11737.7336	4618	5022	5451	541.44784	285.986995	0.048278	0.52901	0.999412	0.571935	2.412243	4.855355	1.177092	1.001918	7.869379	0.000054	0.015419	0.03313	0.141612	345.301732	0.07536	49.206467
163	F	24	0.027051	1.566775	9.430892	0.794587	34618135884	2598	7912	11432.85888	4286	4888	5481	677.887019	346.845229	0.032682	0.52448	0.999405	0.568038	2.442069	5.328651	1.189398	0.991295	14.014453	0.0001	0.028005	0.057379	0.246156	331.10084	0.157146	45.24249
164	F	24	0.022983	1.746607	7.747087	0.768691	34016875214	1654	8706	11136.55697	3969	4503	5130	782.8691	465.318097	0.031824	0.520582	0.999369	0.56538	2.591378	5.506102	1.218123	1.056923	13.299137	0.000112	0.028138	0.060567	0.250359	372.764585	0.36795	46.378327
165	M	25	0.021104	1.848792	6.106094	0.520641	32058310262	1591	6587	10805.40471	3839	4307	4768	607.829157	313.16629	0.035798	0.470304	0.999183	0.526381	3.354619	5.220674	1.41907	1.322401	5.850366	0.000037	0.011863	0.020902	0.113108	189.646341	0.115745	68.442291
166	M	25	0.022159	1.789662	5.05102	0.502591	26150243198	1781	6611	11034.99434	4061	4538	5006	642.516276	372.077147	0.036189	0.481005	0.999196	0.535141	3.299866	5.21295	1.39104	1.344867	6.46984	0.000041	0.012708	0.023983	0.120668	243.824801	0.119296	69.950151
167	F	25	0.024116	1.69144	6.77237	0.620708	35952363553	3048	7907	11138.67476	4749	5196	5693	669.29413	389.876684	0.035411	0.487959	0.999219	0.539983	3.207511	5.27189	1.362459	1.299086	10.121431	0.00006	0.018812	0.037094	0.17051	303.314414	0.121053	58.928763
168	F	25	0.025023	1.650326	7.098591	0.64896	37268501940	2706	7809	11076.96985	4700	5164	5636	650.105431	382.116434	0.036593	0.479293	0.999198	0.53331	3.295072	5.221839	1.393527	1.30481	9.202445	0.000055	0.017246	0.03448	0.157835	271.003425	0.105162	61.18173
169	M	25	0.022858	1.752975	6.769144	0.580361	20053004139	1274	6802	10909.66903	3518	4038	4524	866.688545	513.858205	0.039994	0.515834	0.999371	0.561732	2.581539	5.105468	1.227367	1.032721	7.466898	0.000068	0.01729	0.033974	0.15842	296.254344	0.269583	45.663339
170	M	25	0.022044	1.795859	5.462704	0.558757	30262692126	1692	7518	11360.7215	3910	4439	5010	825.838847	440.342058	0.031273	0.470549	0.99916	0.526011	3.451019	5.424808	1.430572	1.344563	8.265274	0.000058	0.017217	0.031371	0.15821	223.704497	0.177292	55.585236
